# Gut microbiota, circulating inflammatory markers and metabolites, and carotid artery atherosclerosis in HIV infection

**DOI:** 10.1186/s40168-023-01566-2

**Published:** 2023-05-27

**Authors:** Zheng Wang, Brandilyn A. Peters, MacKenzie Bryant, David B. Hanna, Tara Schwartz, Tao Wang, Christopher C. Sollecito, Mykhaylo Usyk, Evan Grassi, Fanua Wiek, Lauren St. Peter, Wendy S. Post, Alan L. Landay, Howard N. Hodis, Kathleen M. Weber, Audrey French, Elizabeth T. Golub, Jason Lazar, Deborah Gustafson, Anjali Sharma, Kathryn Anastos, Clary B. Clish, Robert D. Burk, Robert C. Kaplan, Rob Knight, Qibin Qi

**Affiliations:** 1 Department of Epidemiology and Population Health, Albert Einstein College of Medicine, Bronx, NY USA; 2 Department of Pediatrics, University of California, La Jolla, San Diego, CA USA; 3 Department of Pediatrics, Albert Einstein College of Medicine, Bronx, NY USA; 4 Department of Medicine, Johns Hopkins University, Baltimore, MD USA; 5 Department of Internal Medicine, Rush University Medical Center, Chicago, IL USA; 6 Atherosclerosis Research Unit, Keck School of Medicine, University of Southern California, Los Angeles, CA USA; 7 Hektoen Institute of Medicine, Chicago, IL USA; 8 Department of Internal Medicine, Stroger Hospital of Cook County, Chicago, IL USA; 9 Department of Epidemiology, Johns Hopkins Bloomberg School of Public Health, Baltimore, MD USA; 10 Department of Medicine, State University of New York Downstate Health Sciences University, Brooklyn, NY USA; 11 Department of Neurology, State University of New York Downstate Health Sciences University, Brooklyn, NY USA; 12 Department of Medicine, Albert Einstein College of Medicine, Bronx, NY USA; 13 Department of Obstetrics & Gynecology and Women’s Health, Albert Einstein College of Medicine, Bronx, NY USA; 14 Broad Institute of MIT and Harvard, Cambridge, MA USA; 15 Department of Microbiology & Immunology, Albert Einstein College of Medicine, Bronx, NY USA; 16 Public Health Sciences Division, Fred Hutchinson Cancer Research Center, Seattle, WA USA; 17 Department of Bioengineering, University of California, La Jolla, San Diego, CA USA; 18 Department of Computer Science and Engineering, University of California, La Jolla, San Diego, CA USA; 19 Center for Microbiome Innovation, University of California, La Jolla, San Diego, CA USA; 20 Department of Nutrition, Harvard T.H. Chan School of Public Health, Boston, MA USA

**Keywords:** Gut microbiota, Inflammatory markers, Metabolomics, Atherosclerosis, HIV infection

## Abstract

**Background:**

Alterations in gut microbiota have been implicated in HIV infection and cardiovascular disease. However, how gut microbial alterations relate to host inflammation and metabolite profiles, and their relationships with atherosclerosis, have not been well-studied, especially in the context of HIV infection. Here, we examined associations of gut microbial species and functional components measured by shotgun metagenomics with carotid artery plaque assessed by B-mode carotid artery ultrasound in 320 women with or at high risk of HIV (65% HIV +) from the Women’s Interagency HIV Study. We further integrated plaque-associated microbial features with serum proteomics (74 inflammatory markers measured by the proximity extension assay) and plasma metabolomics (378 metabolites measured by liquid chromatography tandem mass spectrometry) in relation to carotid artery plaque in up to 433 women.

**Results:**

*Fusobacterium nucleatum*, a potentially pathogenic bacteria, was positively associated with carotid artery plaque, while five microbial species (*Roseburia hominis*, *Roseburia inulinivorans*, *Johnsonella ignava*, *Odoribacter splanchnicus*, *Clostridium saccharolyticum*) were inversely associated with plaque. Results were consistent between women with and without HIV. *Fusobacterium nucleatum* was positively associated with several serum proteomic inflammatory markers (e.g., CXCL9), and the other plaque-related species were inversely associated with proteomic inflammatory markers (e.g., CX3CL1). These microbial-associated proteomic inflammatory markers were also positively associated with plaque. Associations between bacterial species (especially *Fusobacterium nucleatum*) and plaque were attenuated after further adjustment for proteomic inflammatory markers. Plaque-associated species were correlated with several plasma metabolites, including the microbial metabolite imidazole-propionate (ImP), which was positively associated with plaque and several pro-inflammatory markers. Further analysis identified additional bacterial species and bacterial *hutH* gene (encoding enzyme histidine ammonia-lyase in ImP production) associated with plasma ImP levels. A gut microbiota score based on these ImP-associated species was positively associated with plaque and several pro-inflammatory markers.

**Conclusion:**

Among women living with or at risk of HIV, we identified several gut bacterial species and a microbial metabolite ImP associated with carotid artery atherosclerosis, which might be related to host immune activation and inflammation.

Video Abstract

**Supplementary Information:**

The online version contains supplementary material available at 10.1186/s40168-023-01566-2.

## Introduction

Emerging evidence has suggested that gut microbiota plays an important role in human atherosclerosis and cardiovascular disease (CVD) [1–5]. Alterations in gut microbiota have been observed in people living with HIV infection [6–8] that has been associated with new formation of carotid artery plaque [9] and increased risk of CVD [10, 11]. Our prior work integrating 16S rRNA sequencing and plasma metabolomics data has identified several gut bacteria genera (e.g., *Fusobacterium*) and related functional capacities in the lipid metabolism (e.g., phospholipase A1 and A2) associated with host circulating lipid metabolites and carotid artery atherosclerosis in women with or at risk of HIV [12]. These data support the hypothesis that gut microbiota might contribute to atherosclerosis and CVD partially through regulating host metabolite profile [1, 4]. However, gut bacterial species associated atherosclerosis and CVD and underlying mechanisms have not been fully understood in the context of HIV infection.

It is known that gut microbiota may shape host immune system that is central to the development of atherosclerosis, and thus, gut microbiota might contribute to atherosclerosis through the modulation of host immune activation and inflammation [5]. Several studies in people without HIV infection [5, 13, 14] and our prior HIV study [12] found that several gut bacterial genera which have potential anti-inflammatory properties (e.g., *Roseburia* and *Odoribacter*) through producing anti-inflammatory molecules (e.g., short chain fatty acids) were lower in people with atherosclerosis or atherosclerotic CVD compared to healthy controls. A small clinical trial of healthy volunteers in Poland also suggested an atheroprotective role of *Lactobacillus plantarum*, a bacterial species known for its anti-inflammatory effects and used as a probiotic [13]. However, most previous human studies on gut microbiota and atherosclerosis/CVD lacked proteomic data on host circulating inflammatory makers [3, 5, 13, 15] and data are even sparse in studies of people living with HIV infection who have chronic inflammation and immune activation.

In the present study, we aimed to identify gut microbiota features focusing on bacterial species and functional components, measured using shotgun metagenomics sequencing, and serum proteomic inflammatory markers, measured by a proteomic platform of 92 proteins, associated with carotid artery plaque in women living with or at risk of HIV from the Women’s Interagency HIV Study (WIHS). In addition, we also related gut microbiota features with host circulating inflammatory markers and metabolites to explore potential mechanisms underlying the relationship between gut microbiota and atherosclerosis in the context of HIV infection.

## Methods

### Study population

The WIHS was a multicenter cohort study of women with or at risk for HIV infection, now continuing as part of the Multicenter AIDS Cohort Study (MACS)-WIHS Combined Cohort Study, and details on study design and methods have been described previously [16–18]. In this study, we included 493 WIHS women whose fecal samples were collected using a home-based self-collection kit [19, 20] during 2017–2019. Among these participants, 320 women underwent carotid artery imaging. We also included 433 women who had proteomic inflammatory marker data from serum samples, and metabolomic/lipidomic data from plasma samples, which were collected during 2017–2019 from the core WIHS visit closest to the time of fecal sample collection (Figure S1).

An expanded description of study population, microbiome sequencing, proteomic inflammation profiling, metabolomic profiling, assessments of carotid artery plaque and HIV variables, and statistical analysis is provided in Supplemental Methods. The study was reviewed and approved by Institutional Review Boards at all participating institutions. All participants provided written informed consent.

### Shotgun metagenomics sequencing

Metagenomics sequencing was performed on DNA extracted from fecal samples collected by FTA card using a shallow-coverage method of shotgun sequencing-based Illumina NovaSeq platforms [21, 22]. The adapters and barcode indices are processed following the iTru adapter protocol [23]. Microbiome bioinformatics analyses and species-level taxonomic assignment were performed using the SHOGUN [24] pipeline and RefSeq database [25], with Bowtie2 [26] as the alignment tool. α-diversity indices (Shannon index, Chao-1 Index and Simpson’s Index) and β-diversity weighted UniFrac distances were calculated using Qiita [27], Metaphlan3 [28], and R phyloseq/vegan packages [29, 30]. Functional components were obtained using SHOGUN and the Kyoto Encyclopedia of Genes and Genomes (KEGG) database [24].

### Proteomic inflammatory markers

Serum proteomics profiling was performed using the Olink® Target Inflammation panel (Olink, Boston, Massachusetts). This panel measures 92 proteins related to immune activation and inflammation by the proximity extension assay. Protein concentrations were reported as normalized protein expression (NPX) units, which are Ct values from the PCR read-out and normalized by the subtraction of values for extension control, as well as an inter-plate control. The scale was shifted using a correction factor (normal background noise) and log^2^ scaled [31, 32]. After quality control, in the current analysis, we included 74 proteomic inflammatory markers which were detected in > 75% of samples.

### Metabolomic/lipidomic profiling

Plasma metabolomic/lipidomic profiling was performed using liquid chromatography-tandem mass spectrometry (LC–MS) at the Broad Institute Metabolomics Platform (Cambridge, Massachusetts) as previously described [33–35]. We included 211 lipids and 167 polar metabolites in the current analysis, and all metabolites had coefficient variation < 30% and missing rate < 20%. Metabolites with missing data (under detectable levels) were imputed with ½ the minimum values for a given metabolite.

### Carotid artery plaque ascertainment

High-resolution B-mode carotid artery ultrasound was used to image 8 locations in the right carotid artery of participants: the near and far walls of the common carotid artery, carotid bifurcation, and internal and external carotid artery [36], using standardized protocols [36, 37]. We defined a focal plaque as an area with localized intima-media thickness > 1.5 mm in any of the 8 imaged carotid artery locations [38].

### Assessments of HIV infection and other variables

HIV status was ascertained by enzyme-linked immunosorbent assay and confirmed by Western blot. HIV-specific parameters included CD4 + T cell counts, HIV-1 viral load, and detailed information on specific classes of ART drugs (protease inhibitors, nonnucleoside reverse transcriptase inhibitors, and nucleoside reverse transcriptase inhibitors) [39]. Conventional CVD risk factors included body mass index (BMI), systolic blood pressure (SBP), diastolic blood pressure (DBP), triglycerides, total cholesterol, low-density lipoprotein cholesterol, high-density lipoprotein cholesterol, fasting glucose, hemoglobin A1c, anti-cholesterol, and anti-hypertensive medication [40]. Assessments of other variables are described in Supplemental Methods.

### Statistical analysis

We first examined associations of gut microbial species with carotid artery plaque in 320 women, using linear discriminant analysis effect size (LefSe) [41]. Logistic regression was performed to examine multivariable-adjusted associations between gut microbial species (central log ratio (CLR) transformation was conducted for the species level taxonomic abundance) and carotid artery plaque, adjusting for age, race/ethnicity, study site, antibiotic use, income, education, BMI, alcohol, smoking status, HIV status, and ART use. In addition, we also applied Analysis of Composition of Microbiomes (ANCOM2) [42] to identify gut bacterial species associated with plaque, adjusting for the aforementioned covariates. We excluded species present in < 20% of the population or with average relative abundance < 0.001%. We controlled the false discovery rate (FDR) at 10%. Multivariable linear regression was used to examine associations between microbial functional enzymes (CLR-transformed) and plaque. An enrichment test was performed for 1634 annotated enzymes at EC level III enzyme category, with FDR < 0.10 as the cutoff. Partial Spearman correlations were performed among enzymes and plaque-related species adjusting for the aforementioned covariates.

Partial least squares discriminant analysis (PLSDA) [43] with loading scores was used to identify proteomic inflammatory markers associated with carotid artery plaque and their contribution to each PLSDA component among 290 women including 75 plaque cases. Logistic regression was used to examine associations of PLSDA components and individual inflammatory markers (inverse normal-transformed) with plaque, adjusting for the aforementioned covariates. Associations of plaque-related species with proteomic components and individual proteomic inflammatory markers were estimated by Spearman correlation among 426 women. We further adjusted for inflammatory marker profiles (PLSDA components) in the logistic regression to examine whether the association between microbial species and plaque could be partially explained by inflammatory markers.

We examined associations of 6 plaque-associated species with 211 lipids and 167 polar metabolites using Spearman correlation (*n* = 426, Figure S1). Logistic regression was used to examine associations between selected microbiota-correlated metabolites and plaque (*n* = 290). We explored correlations among these microbiota-correlated metabolites and proteomics profiles (*n* = 433). We further adjusted for microbial metabolites and inflammatory marker profiles in the logistic regression to examine whether the association between microbial species and plaque could be partially explained by these metabolites and inflammatory markers (*n* = 285).

Spearmen correlation was used to identify gut bacterial species associated with plasma ImP (*n* = 426). We then included all the 138 ImP-correlated species in the same linear regression model (mutual adjustment) to further examine independent associations.

To derive a global measure representing the overall gut bacterial species associated with ImP, we calculated a gut microbiota score based on the sum of the weighted INT-CLR (inverse normal-central log ratio) transformed abundance of the 17 species independently associated with ImP (weighted by beta-coefficient estimated in the mutual adjustment linear regression model). Associations of the gut microbiota score and 17 individual species with ImP and plaque status were examined using multivariable linear and logistic regression, respectively. We then used linear regression to examine associations among levels of functional enzyme *hutH*, the gut microbiota score, and plasma ImP. We also explored the correlations of the GMB score and 17 ImP-associated species with functional enzyme *hutH* and proteomic profiles.

Statistical analyses were performed using R 4.0.2. unless otherwise stated. To explore potential HIV-specific results, the stratified analyses were also conducted in women with and without HIV separately.

## Results

### Gut microbiota and carotid artery plaque

Table S1 shows characteristics of 320 participants (236 without plaque and 84 with plaque), who were included in the analysis of the association between gut microbiota and carotid artery plaque. Compared with those without plaque, participants with plaque were older and more likely to have higher levels of CVD risk factors (e.g., higher blood pressure). The majority of women with HIV reported ART use (92% and 93%, respectively, in participants with/without plaque) and had undetectable HIV-1 viral load (≤ 20 copies/mL, 73% and 78%, respectively). In line with the overall WIHS participants [17], sociodemographic characteristics among participants with and without HIV were generally similar (data not shown). We observed that 76.4% of women on ART (152 out of 199) had undetectable HIV-1 viral load (≤ 20 copies/mL) and then compared demographic and other characteristics between women on ART with and without viral suppression (Table S2). We did not find significant differences except lower BMI in women without viral suppression compared to those with viral suppression. Since the cutoff of viral suppression in our WIHS cohort (≤ 20 copies/mL) was relatively stringent [17], we also evaluated viral suppression using ≤ 200 copies/mL as the cutoff [44, 45] and 92.0% (183 out of 199) of women on ART had viral suppression.

**Fig. 1:**
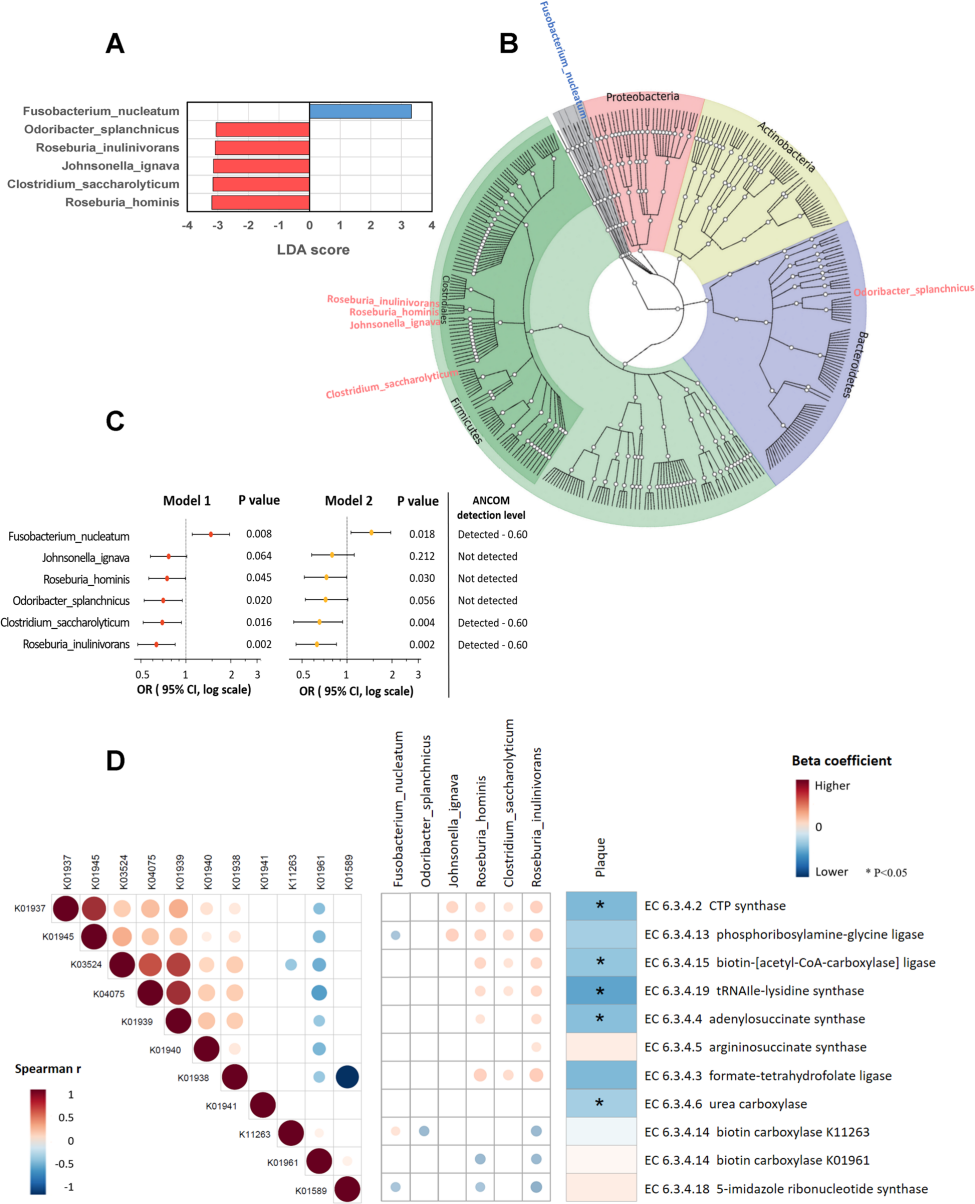
Differentially abundant species according to carotid artery plaque status.** A** Taxonomic linear discriminative analysis (LDA) effect size (LefSe) analysis by carotid artery plaque status. **B** Integrated phylogenetic tree. Taxa from inner to outer circle represent bacteria kingdom to species level. Blue and red font represent positive and negative associations between species and plaque, respectively. **C** Associations between species and plaque status. Data are odds ratios (ORs) and 95% confidence intervals (CIs) for carotid artery plaque per standard deviation increment of CLR transformed abundance of gut bacterial species, adjusted for age, race, study site, antibiotics use, income, education, BMI, alcohol, smoking status, HIV status, and ART use (model 1) and further adjusted for blood pressure, total cholesterol, high-density lipoprotein cholesterol, antihypertensive medication use, and lipid-lowering medication use (model 2). In addition, we also show ANCOM2 detection level of each species, adjusting for aforementioned covariates in model 1. **D** Associations between gut microbiota functional enzymes, plaque-associated bacteria species, and carotid artery plaque status. The Spearman correlation heatmap include 11 microbial functional enzymes under carbon–nitrogen ligases category and the 6 plaque-associated bacterial species. Associations between the 11 microbial functional enzymes and carotid artery plaque status were estimated by linear regression models after adjustment for age, race, study site, antibiotics use, income, education, BMI, alcohol, smoking status, HIV status, and ART use

We did not find significant associations of *α*-diversity indices (Shannon index, Chao-1 Index, and Simpson’s Index) (all *P* > 0.05, Figure S2A) or *β*-diversity (measured using weighted UniFrac distances) (Figure S2B; *R*^2^ < 0.1, *P* > 0.05, PERMANOVA analysis) with plaque. In individual taxonomy analyses, LefSe indicated that carotid artery plaque was associated with enriched *Fusobacterium nucleatum* and depleted *Roseburia hominis*,* Roseburia inulinivorans*,* Odoribacter splanchnicus*, *Clostridium saccharolyticum*, and *Johnsonella ignava* (all LDA score > 3, Fig. 1A). An integrated phylogenetic tree indicated that four of the species inversely associated with plaque belong to the same order *Clostridiales* (i.e.,* R. hominis*,* R. inulinivorans*,* J. ignava*,* C. saccharolyticum*), within the phylum Firmicutes (Fig. 1B), while *F. nucleatum* and *O. splanchnicus* belong to phylum Fusobacteriota and Bacteroidetes, respectively. These two species, *F. nucleatum* and *O. splanchnicus*, belong to two genera, *Fusobacterium* and *Odoribacter*, which have been identified to be associated with plaque in our prior work using 16S rRNA data [12]. In this study, our shotgun metagenomics showed similar results on four plaque-associated bacterial genera identified in our previous analysis using 16S data (Figure S3).

We then focused on these plaque-associated species in subsequent analyses. After multivariable adjustment, there were significant differences in the CLR-transformed abundance of *F. nucleatum*,* R. hominis*,* R. inulinivorans*,* O. splanchnicus*, and *C. saccharolyticum* between women with and without plaque (Fig. 1C). Higher levels of *F. nucleatum* were associated with elevated odds of carotid artery plaque (OR [95% CI] = 1.47 [1.10, 1.96], per SD increment in CLR-transformed abundance) while higher levels of *R. inulinivorans*,* R. hominis*,* O. splanchnicus*, and *C. saccharolyticum* were associated with lower odds of plaque (OR [95% CI] = 0.64 [0.48,0.85], 0.75 [0.56,0.99], 0.71 [0.53,0.95], and 0.70 [0.52,0.93], respectively) (all *P* < 0.05). These associations were attenuated but remained significant (all *P* < 0.05) after further adjustment for conventional CVD risk factors (Fig. 1C), except for *O. splanchnicus *(*P* = 0.056).* J. ignava* showed a marginally significant association with carotid artery plaque (*P* = 0.064), and the association was abolished after further adjustment for conventional CVD risk factors (*P* = 0.212). In addition, we also applied ANCOM2 to identify bacterial species associated with carotid artery plaque. Consistently, three species (*F. nucleatum*,* R. inulinivorans*, and *C. saccharolyticum)* were also identified to be associated with carotid artery at a detection level of 0.6, and no additional species were detected.

We did not find many significant correlations between these plaque-associated species with conventional CVD risk factors, except for a moderate inverse correlation between *O. splanchnicus* and SBP (*r* =  − 0.17, *P* = 0.009) and weak correlations of *C. saccharolyticum* with BMI and TG (*r* = 0.15, *P* = 0.011; *r* =  − 0.13, *P* = 0.041, respectively) (Figure S4). The observed moderate correlation of *O. splanchnicus* with blood pressure might partially explain the attenuation of the association between *O. splanchnicus* and plaque after further adjustment for CVD risk factors.

We did not observe significant associations of these six plaque-associated species with HIV serostatus and HIV-related factors (CD4^+^ T cell count, HIV viral load, and ART use), after adjustment for demographic, behavioral, and clinical factors (all *P* > 0.05, Tables S3 and S4). We then focused on women on ART and the relative abundance of the six plaque-associated species were similar between those with and without viral suppression (Figure S5). We did not observe significant associations between these six plaque-associated species and HIV viral suppression, after controlling for the aforementioned covariates (Table S5).

In LefSe analyses stratified by HIV serostatus, 5 of 6 plaque-associated species that were identified in the overall sample were also detected in women with HV (Figure S6A), while 3 of these 6 species were detected in women without HIV which might be due to a relatively small sample size in this group (Figure S6B). We found two additional plaque-associated species (*Fournierella massiliensis* and *Collinsella aerofaciens*) that met nominal statistical significance levels only in women with HIV. However, the associations between these gut bacterial species and carotid artery plaque were generally consistent between women with and without HIV, and no effect modification by HIV serostatus was observed (all *P* for interaction > 0.05, Table S6).

We next examined associations between gut microbiome functional components and carotid artery plaque. After controlling for demographic, behavioral, clinical variables, and HIV-related variables, we identified 164 enzymes associated with plaque (all *P* < 0.05). We then performed enrichment tests at EC enzyme category level III and found that plaque was associated with the enrichment of enzymes belonging to specific categories (e.g., EC 6.3.4 carbon–nitrogen ligases; *P* = 0.002, FDR < 0.1, Table S7). In our study, 11 enzymes were detected under the carbon–nitrogen ligases category, 7 of which were positively correlated with each other (Fig. 1D). These 7 enzymes were also positively associated with at least one of the beneficial species under *Clostridiales* (*R. hominis*,* R. inulinivorans*,* J. ignava*, and *C. saccharolyticum*), and 4 of these enzymes were also inversely associated with plaque, after multivariate adjustment (Fig. 1D). We also found that plaque was associated with potential enrichment of enzymes EC 3.1.1 carboxylic-ester hydrolases (*P* = 0.046, Table S7) which has been identified in our previous analysis using 16S data [12].

### Proteomic inflammatory markers and carotid artery plaque

**Fig. 2:**
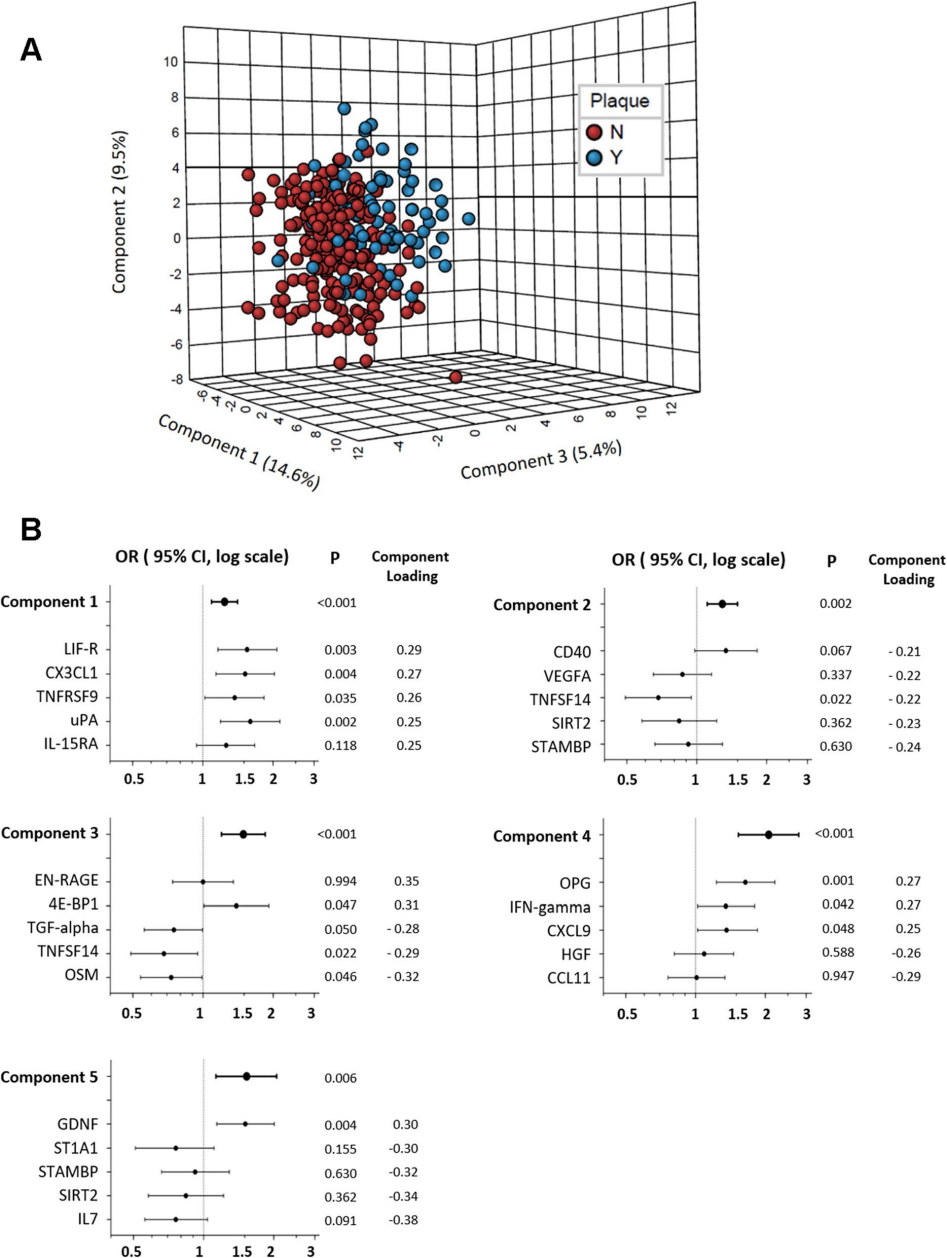
Serum proteomic inflammatory markers and carotid artery plaque. **A** Serum proteomic inflammatory markers and carotid artery plaque: Partial Least Square-Discriminant Analysis (PLSDA) plot by plaque status. **B** The major contributors of each PLSDA PCs and their associations with carotid artery plaque. Data are odds ratios (ORs) and 95% confidence intervals (CIs), adjusted for age, race, study site, antibiotics use, income, education, BMI, alcohol, smoking status, HIV status, and ART use

Partial Least Square-Discriminant Analysis (PLSDA) of all 74 inflammatory markers revealed a distinction between groups with and without plaque, although these two groups were not fully separated (Fig. 2A). The top five out of the eight PLSDA PCs of proteomic inflammatory markers showed positive associations with plaque, after controlling for demographic, behavioral, clinical variables, and HIV-related variables (OR = 1.24 [95% CI, 1.09, 1.41], 1.29 [1.11, 1.50], 1.49 [1.20, 1.85], 2.07 [1.53, 2.79], and 1.53 [1.13, 2.06], respectively) (Fig. 2B and Table S8).

We then examined associations of individual inflammatory markers, which were identified as the top 5 major contributors of each PLSDA PC, with carotid artery plaque. Each component had different top contributors, except for PC2, which shared TNFSF14 with PC3 and shared SIRT2 and STAMBP with PC5. Since all 5 PCs were positively associated with plaques, individual contributors of these PCs which showed positive PC loading scores were generally positively associated with plaque, while individual contributors which showed negative PC loading scores were generally inversely associated with plaque, although some of them did not reach statistically significance (12 out of 22 with *P* < 0.05). For example, 4 of 5 PLSDA PC1 major contributors, which had positive PC loading scores, also showed positive associations with plaque significantly. These were Urokinase-type plasminogen activator (uPA), leukemia inhibitory factor receptor (LIF.R), Fractalkine (CX3CL1), and tumor necrosis factor receptor superfamily member 9 (TNFRSF9).

In stratified analyses by HIV serostatus, we observed consistent results across strata (Table S8). No effect modification by HIV serostatus was observed (all *P* for interaction > 0.05).

### Carotid artery plaque-associated microbial species and serum inflammatory markers

**Fig. 3:**
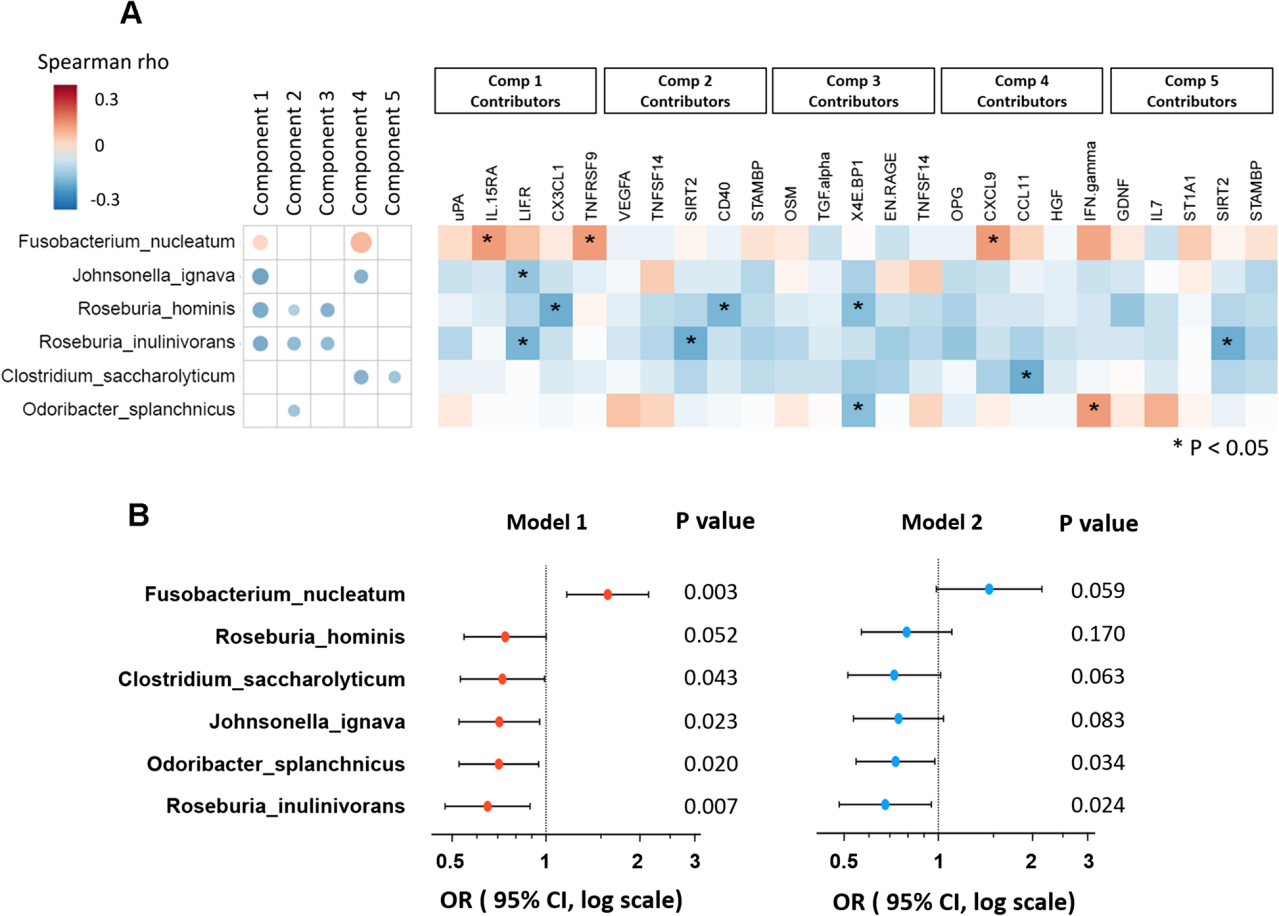
Associations of plaque-associated bacterial species with host proteomic inflammatory markers.** A** Microbial species, proteomics PLSDA PCs, and their major contributors. **B** Associations between microbial species and plaque status, adjusted for proteomics profiles. Data are odds ratios (ORs) and 95% confidence intervals (CIs) for carotid artery plaque per standard deviation increment of CLR transformed abundance of gut bacterial species, adjusted for age, race, study site, antibiotics use, income, education, BMI, alcohol, smoking status, HIV status, and ART use (model 1) and further adjusted for top 5 proteomics PLSDA PCs (model 2). Abbreviations: PLSDA, partial least square-discriminant analysis

We next examined associations between plaque-associated gut microbial species and serum inflammatory markers among 426 participants who had both shotgun metagenomics and proteomics data available. *F. nucleatum*, which was positively associated with plaque, showed positive correlations with proteomic PLSDA PC1 and PC4 **(**Fig. 3A**)**, while potentially beneficial microbial species, which were inversely associated with plaque, showed negative correlations with at least one proteomics PLSDA PC. For example, both *R. hominis* and *R. inulinivorans* showed negative correlations with proteomic PLSDA PC1, PC2, and PC3. Correlations between plaque-associated microbial species and individual inflammatory markers which served as major contributors of these PLSDA PCs were also observed.

The observed associations between gut microbial species and carotid artery plaque were attenuated after further adjustment for the top 5 proteomic PLSDA PCs **(**Fig. 3B**)**. These results suggested that the proteomic inflammatory markers might partially explain the association between gut microbiota and plaque.

### Plasma metabolomic profiles, gut microbiota, and serum inflammatory markers, in relation to carotid artery plaque

We further examined associations between plaque-associated gut microbial species and plasma lipidomic/metabolomic profiles. As shown in Figure S7A and S7B,* F.nucleatum*, which was positively associated with plaque, was significantly correlated with 70 polar metabolites (out of total 167 polar metabolites) and 46 lipids (out of total 211 lipids; all FDR *P* < 0.1). These results are highly consistent with our previous analyses using 16S data [12]. For microbial species inversely associated with plaque, we only found a small number of metabolites and lipids correlated with these microbial species (Figure S7A, S7B**)**. However, interestingly, among five polar metabolites which were correlated with at least one beneficial species, two are known microbially produced metabolites (imidazole propionate (ImP) [46, 47], L-Urobilin [48]), and 3-hydroxyhippuric acid and ornithine are also related to microbial metabolism [49, 50].

**Fig. 4:**
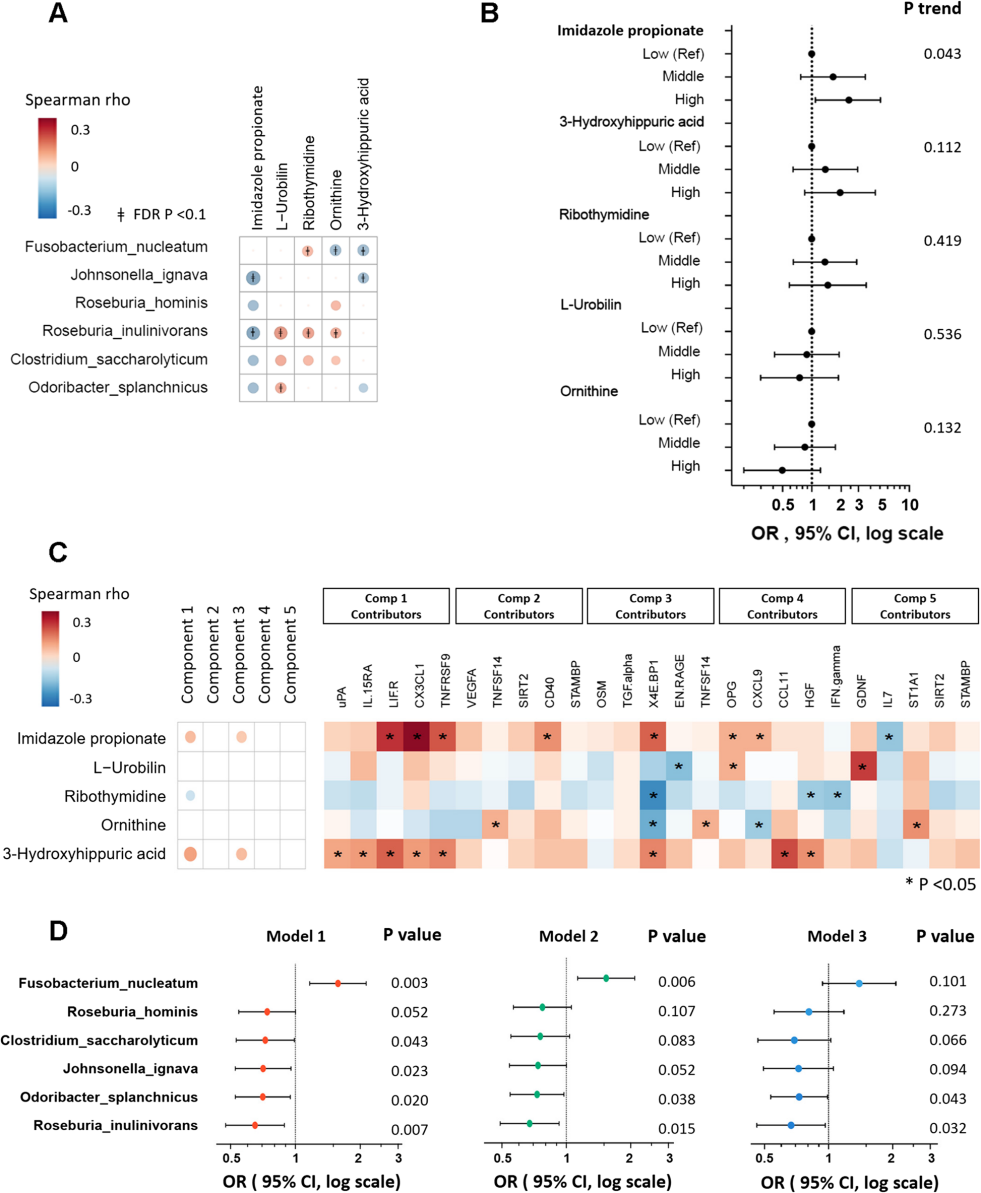
Plasma metabolomic profiles, gut microbiome, and serum proteomic inflammatory markers, in relation to carotid artery plaque.** A** Plaque-associated microbial species and polar metabolites. **B** Associations between microbial-associated metabolites and carotid artery plaque, adjusted for age, race, study site, antibiotics use, income, education, BMI, alcohol, smoking status, HIV status, and ART use. Levels: low, quartile 1 of the metabolite; medium, quartile 2 and 3; high, quartile 4. **C** Microbial-associated metabolites, proteomics PLSDA PCs, and their major contributors. **D** Associations between microbial species and plaque status, adjusted for metabolomic profiles. Data are odds ratios (ORs) and 95% confidence intervals (CIs) for carotid artery plaque per standard deviation increment of CLR transformed abundance of gut bacterial species, adjusted for age, race, study site, antibiotics use, income, education, BMI, alcohol, smoking status, HIV status, and ART use (model 1); further adjusted for Imidazolepropionic acid (model 2); and further adjusted for top 5 proteomics PLSDA PCs (model 3). Abbreviations: Ref, reference group; PLSDA, partial least square-discriminant analysis

We then examined associations of these 5 polar metabolites with carotid artery plaque. In particular, ImP, a microbial metabolite from histidine [46, 47], was inversely associated with all 5 potentially beneficial microbial species (Fig. 4A) and positively associated with carotid artery plaque (Fig. 4B, P= 0.043). ImP was also positively correlated with proteomic PLSDA PC1 and PC3 and a number of individual inflammatory markers including those major contributors for PC 1 (LIF.R, CX3CL1, and TNFRSF9) or PC3 (4E-BP1), and several others (CD40, OPG and CXCL9) (Fig. 4C). In addition, we found a trend of positive association between carotid artery plaque and 3-hydroxyhippuric acid (*P* = 0.11), which was also known as a microbial-related metabolite [49]. 3-hydroxyhippuric acid was positively correlated with proteomic PLSDA PC1 and PC3, and a number of individual inflammatory markers. In stratified analyses by HIV status, we observed consistent associations between these metabolites and plaque across strata (Table S9), without effect modifications by HIV serostatus (all *P* for interaction > 0.05).

In 285 WIHS participants with gut microbiome, proteomics, metabolomics, and plaque data, the association between 6 gut bacterial species and plaque were attenuated after further adjustment for plasma ImP levels, especially for those potentially beneficial species. Most of these associations were further attenuated after further adjustment for the top 5 proteomic PLSDA PCs (Fig. 4D**)**. The association between these 6 specific microbial species and plaque did not change materially after further adjustment for plasma 3-hydroxyhippuric acid levels (Figure S8). These results suggest that circulating levels of ImP and related inflammatory markers could partially explain the associations between these gut bacterial species and plaque.

### ImP, ImP-related microbial species, and carotid artery plaque

**Fig. 5:**
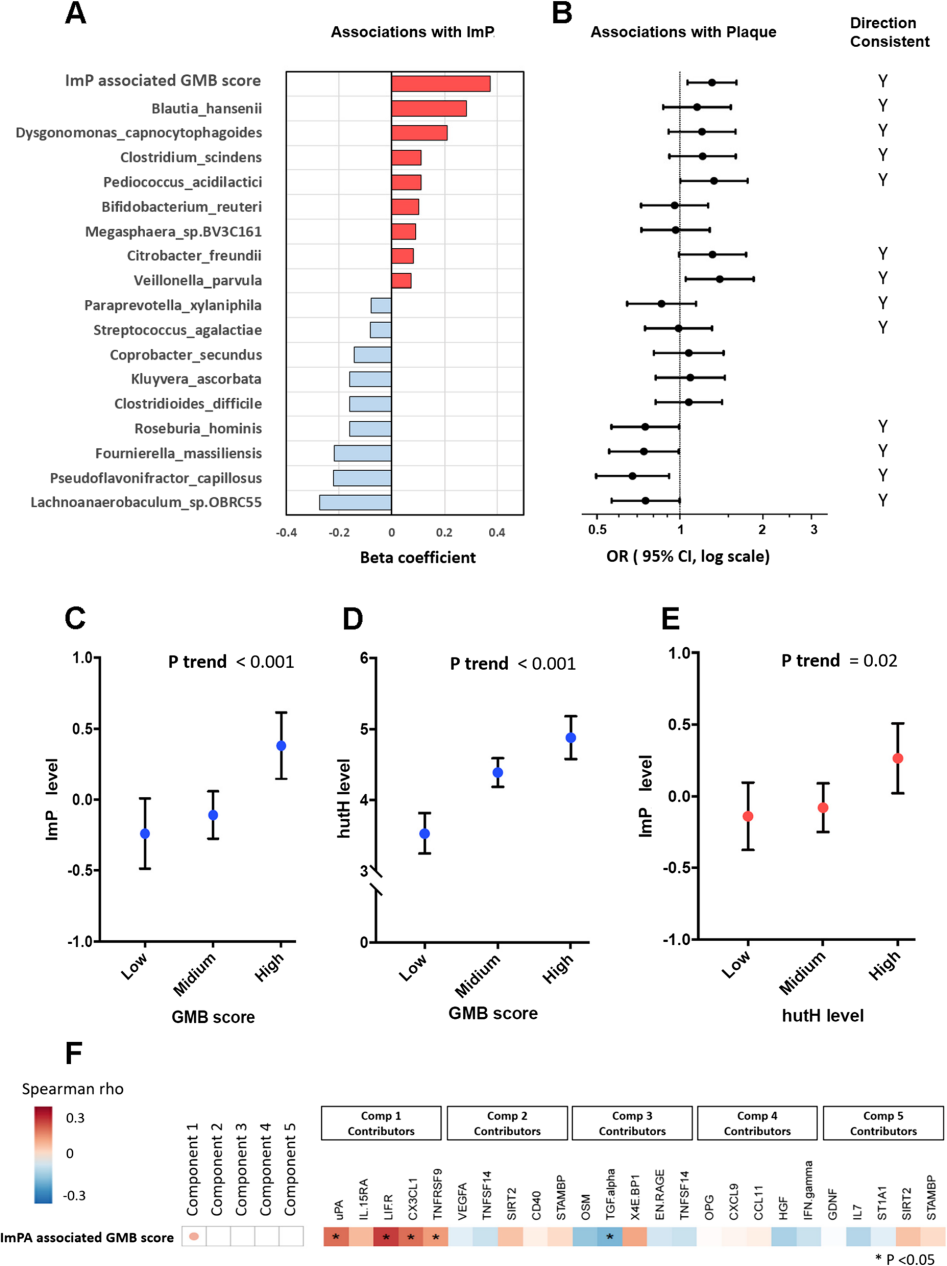
ImP, ImP-related microbial species, functional components, and serum inflammatory markers, in relation to carotid artery plaque. **A** Associations between ImP, gut microbiota (GMB) score, and individual microbial species, after adjustment for demographic, behavioral, clinical, and HIV-specific variables. The beta coefficients for individual microbial species were estimated in conditional analysis regression models, with mutual adjustment for other ImP-related microbial species. **B** Associations between the identified ImP-associated microbial species, GMB score, and carotid artery plaque. Data are odds ratios (ORs) and 95% confidence intervals (CIs) for carotid artery plaque per standard deviation increment of CLR transformed abundance of gut bacterial species, adjusted for demographic, behavioral, clinical, and HIV-specific variables. **C** Plasma ImP and **D** gut microbial enzyme *hutH*, according to the levels of ImP associated GMB score (low, quartile 1 of GMB score; medium, quartiles 2 and 3; high, quartile 4). Data are adjusted means and 95% CIs estimated from regression after adjustment for demographic, behavioral, clinical, and HIV-specific variables. **E** Plasma ImP according to the levels of gut microbial enzyme hutH (low, quartile 1 of *hutH*; medium, quartiles 2 and 3; high, quartile 4). Data are adjusted means and 95% CIs estimated from regression after adjustment for demographic, behavioral, clinical, and HIV-specific variables. **F** ImP-associated GMB score, proteomics PLSDA PCs, and their major contributors. Abbreviations: ImP, imidazole propionate; GMB, gut microbiota; PLSDA, partial least square-discriminant analysis

Since the circulating ImP was positively associated with carotid artery plaque (Fig. 4B, *P*= 0.043), and it was also inversely associated with all five plaque-associated beneficial microbial species, we then focused on this microbial metabolite [46, 47] and aimed to identify more gut microbial species associated with ImP in our study. A total of 138 out of all 316 gut microbial species were significantly correlated with plasma ImP levels (FDR < 0.1) (Table S10). We then included all 138 bacteria species in the same linear regression model, and this analysis suggested17 bacterial species independently associated with ImP, including 8 species positively and 9 species negatively associated with ImP (Fig. 5A and Table S11). Most of the directions of these associations (12 of 17) were in line with the directions of their associations with carotid artery plaque (Fig. 5B). An ImP-associated gut bacterial score was significantly associated with higher plasma ImP levels and higher odds of plaque (OR = 1.31 [95% CI, 1.07, 1.61]), after controlling for demographic, behavioral, clinical, and HIV-specific variables.

The microbial functional enzymes histidine ammonia lyase (EC:4.3.1.3, encoded by the *hutH* gene) and urocanate reductase (EC:1.3.99.33, encoded by the *urdA* gene) are known key enzymes in reactions producing ImP from histidine [46, 47]. We explored potential contributions of the ImP-associated species to these enzymes, especially *hutH*, since *urdA* was not detected in our shallow shotgun sequencing data. We found that 3 ImP-positively-associated species were positively correlated with *hutH* (*Blautia hansenii*,* Bifidobacterium reuteri*, and *Veillonella parvula*) and 5 ImP-inversely associated species were inversely correlated with *hutH* **(**Table S12). Our genomic analyses provided further evidence supporting the presence of *hutH* gene on the representative genomes of *Blautia hansenii* and *Bifidobacterium reuteri* which were positively associated with both ImP and *hutH* level in our study (Table S13). In addition, we found the presence of the reference *urdA* gene sequence on the genome of *Clostridium scindens* which was not associated with *hutH* but positively associated with ImP, which might partially explain its positive association with ImP (Table S13). As expected, we did not find the presence of *hutH* or *urdA* within the genomes of those microbial species which were inversely associated with ImP.

Both microbial *hutH* levels and plasma ImP levels increased with higher gut bacterial score (Fig. 5C, D), which are in line with the positive association between microbial *hutH* levels and plasma ImP levels (Fig. 5E). In addition, we also found that the ImP-associated gut bacterial score was positively correlated with proteomics PLSDA PC1 and its 4 major contributors, uPA, LIF.R, CX3CL1, and TNFRSF9, and inversely correlated with TGF.alpha (Fig. 4F).

## Discussion

In this study of women living with or at risk of HIV infection, our current analysis using shotgun metagenomics data extended our prior findings based on 16S data [12] to show multiple gut bacterial species and related inflammatory markers and metabolites associated with carotid artery atherosclerosis. We identified two gut bacterial species associated with carotid artery plaque, *F. nucleatum* and *O. splanchnicus*, which belong to two previously reported genus, *Fusobacterium* and *Odoribacter*, respectively [12]. Moreover, by integrating data on serum proteomic inflammatory markers, this study revealed potential inter-relationships among these microbial species, host inflammation and immune activation, and atherosclerosis. In particular, we identified that gut *F. nucleatum* was positively associated with several serum proteomic markers which might be involved in host inflammation and immune activation related to bacterial infection (e.g., CXCL9, TNFRSF9) [51–54]. Of note, CXCL9 is a known pro-inflammatory chemokine [53] and contributes to host antimicrobial activity against many pathogenic bacteria [55, 56]. CXCL9 is highly expressed in response to infection of some intestinal pathogens (e.g., *Citrobacter rodentium*) [55, 56], though it is unknown whether expression of CXCL9 can also be induced by *F. nucleatum*, a proinflammatory pathogenic bacteria which is related to inflammatory bowel disease [57]. TNFRSF9 is also a known proinflammatory marker in tumor necrosis factor receptor superfamily which is involved in the formation of atherosclerotic plaques through T cell activation [58], and circulating levels of TNFRSF9 could be affected by the alternations of gut microbiota [59]. Our further analysis suggested that the association between *F. nucleatum* and carotid artery plaque might be partially explained by serum inflammatory markers. This supports the hypothesis that gut microbiota may contribute to atherosclerosis and CVD partially through host inflammation and immune activation pathway [5].

This study also identified additional four gut bacterial species inversely associated with atherosclerosis (i.e., *R. hominis*,* R. inulinivorans*,* J. ignava*,* C. saccharolyticum*) in women living with or at risk of HIV infection. All of these four bacterial species belong to the same order *Clostridiales* in the phylum *Firmicutes*. In line with our results, gut *Roseburia* species have been reported to be favorably associate with atherosclerosis and CVD in animal models [60, 61] and in human non-HIV populations [3, 5], potentially related to the production of microbial metabolites (e.g., SCFAs) which may have anti-inflammatory effects and beneficial effects on cardiometabolic health [14, 61]. Our findings provide further evidence supporting the beneficial role of gut *Roseburia* species in atherosclerosis and CVD partially through anti-inflammatory effects. In this study, the two *Roseburia* species were inversely associated with several serum inflammatory markers, including CX3CL1 and LIF-R, which both have a potential role of inflammatory mediators in the formation of atherosclerotic plaques [62, 63]. Consistently, serum levels of both CX3CL1 and LIF-R were positively associated with carotid artery plaque in this study. However, less is known how these serum inflammatory markers might be related to gut microbiota, although serum CX3CL1 has been related to bacterial clearance in sepsis [64].

Another major finding of this study is that all five potentially beneficial species were inversely associated with plasma levels of Imp, a known microbial metabolite derived from histidine [46], which were associated with high blood pressure [65], insulin resistance and type 2 diabetes [46, 47] in previous studies, and carotid artery plaque in this study. Previous study revealed that ImP is a microbiota-dependent metabolite [46]; however, the specific taxa involved in ImP production are not fully elucidated. Our further analysis using a mutual adjustment model identified 8 microbial species independently associated with higher plasma ImP levels, including *Clostridium scindens* and *Veillonella parvula* which have been previously reported to be associated circulating ImP levels [47]. For these ImP-associated species, most of the directions of their associations with ImP were in line with the directions with plaque. These ImP-associated species might have a potentially cumulative effect on the development of plaque as we found that a gut bacterial score based on these ImP-associated species was significantly associated with higher plasma ImP levels and higher odds of plaque. Furthermore, some of these ImP-associated species were also positively correlated with *hutH* (e.g., *Blautia hansenii* and *Bifidobacterium reuteri*), a key enzyme in ImP production from histidine [46, 47], and the presence of *hutH* gene on the representative genomes of these species was confirmed by our microbial genomic analysis. Although we did not detect the other key enzyme related to ImP production, *urdA*, in our study sample, which might be due to its relatively lower abundance compared to *hutH* [46, 47], we found the *urdA* gene sequence on the reference genome of *Clostridium scindens*, a known species that can produce ImP [46]. In addition, ImP has been suggested to contribute to type 2 diabetes by promoting low-grade inflammation [46, 47]. We extend this previous study to show that both serum ImP levels and the ImP-associated GMB score were associated with serum inflammatory markers, including CX3CL1, TNFSRF9, and LIF-R which are in the immune activation and inflammation pathways related to atherosclerotic plaques [58, 62, 63]. Taken together, these data suggest that an unfavorable gut bacterial profile, with high abundance of ImP producers (e.g., *Clostridium scindens*) and low abundance of beneficial bacteria (e.g., *Roseburia* species), might be associated with increased circulating plasma ImP levels that may contribute to atherosclerosis and CVD by modulating host immune activation and inflammation.

In addition, we also found another microbiota-related metabolite, 3-hydroxyhippuric acid, which showed a trend of positive association with carotid artery plaque and also positive associations with several inflammatory markers. 3-hydroxyhippuric acid is a microbial aromatic acid metabolite derived from dietary polyphenols and flavonoids [66] through microbial metabolism [49]. However, our data indicated that the associations between the identified gut bacterial species and carotid artery plaque might be independent of circulating 3-hydroxyhippuric acid levels, since further adjustment for 3-hydroxyhippuric acid did not materially change these associations.

To the best of our knowledge, this is the first study integrating the gut microbiome with host circulating proteomic inflammatory markers and metabolites in relation to subclinical atherosclerosis in the context of HIV infection. A number of previous human studies, mostly based on amplicon 16S rRNA sequencing, have linked gut microbiota to atherosclerosis [2, 3, 5] and CVD [4, 67, 68] in non-HIV populations and yielded various results. Multiple gut bacterial taxa have been reported to be associated with atherosclerosis, but only few bacterial taxa were confirmed across different studies (e.g., *Roseburia*) [3, 5]. Inconsistencies among these studies might be due to the use of various CVD outcomes, relatively small sample sizes in most studies, and differences in study design and population characteristics. Consistent with previous findings in non-HIV populations [3, 5], this study also suggested a potential beneficial role of gut *Roseburia* in atherosclerosis and CVD among women with or at risk of HIV infection. However, this study found little evidence for effect modification by HIV status on the relationship between gut microbiota and carotid artery plaque or any HIV-specific findings, although HIV infection has been associated with increased risk of carotid artery plaque [10], as well as alterations of gut microbiome [6–8].

This study has several limitations. The ability to assess effect modification by HIV infection was underpowered due to the limited sample size, particularly for women without HIV. Due to limitations of shallow shotgun metagenomics, some known low abundant functional components such as *urdA* were not detected. The associations of gut microbiota with host serum inflammatory markers, plasma metabolomic profiles, and carotid artery plaque were examined in a cross-sectional dataset of women with or at high risk of HIV. Replication of our findings in both women and men from other HIV cohorts would provide further validation. Given the nature of this observational study, causal inference cannot be established without further evidence.

In summary, this study identified altered gut microbial species, serum inflammatory markers, and plasma metabolites in relation to carotid artery atherosclerosis in women with or at risk of HIV. The enrichment of *F. nucleatum*, which positively associated with multiple inflammatory markers, was associated with atherosclerosis, while two *Roseburia* species, which exhibited potential anti-inflammatory capacity, were inversely associated with atherosclerosis. Furthermore, the microbially produced metabolite ImP and related gut bacterial profile were associated with serum inflammatory markers and carotid artery atherosclerosis. This study provides new information to help better understand the interrelationship among the gut microbiome, host inflammation and metabolism, and CVD in the context of HIV infection. Further investigations are warranted to evaluate the therapeutic potential of anti-inflammation by modulating the gut microbiota and related metabolites in the prevention of atherosclerosis and CVD.

## Supplementary Information

**Additional file 1:** Supplementary methods. Supplementary results. **Figure S1.** Number of participants for omics measurements. **Figure S2.** Microbial community level assessment. (A) α-diversity analyses by carotid artery plaque status. (B) Principal coordinates analysis (PCoA) of β-diversity using weighted UniFrac distances. **Figure S3.** Comparison of Amplicon 16S rRNA Sequencing and Shotgun Metagenomics Sequencing: plaque-associated bacterial genera. **Figure S4.** Spearman correlations between plaque-associated bacterial species and traditional CVD risk factors. **Figure S5.** Average relative abundance of plaque-associated species by viral suppression, among women on ART. **Figure S6.** Differentially abundant species according to Carotid Artery Plaque status, stratified by HIV infection. **Figure S7.** Correlations between plaque-associated bacterial species and plasma metabolites (A) polar metabolites (B) lipids. **Figure S8.** Associations between microbial species and plaque status, adjusted for metabolomic profiles. **Table S1.** Characteristics of study participants. **Table S2.** Characteristics of women on ART, by viral load. **Table S3.** Associations of gut microbial species with HIV status. **Table S4.** Associations of gut microbial species with HIV-specific variables, among women with HIV. **Table S5.** Associations of gut microbial species with Viral load, among women on ART. **Table S6.** Associations of bacterial species with Plaque status, stratified by HIV status. **Table S7.** GMB functional enzymes and carotid artery plaque: enrichment test. **Table S8.** Associations of proteomic inflammatory markers PLSDA PCs with plaque status, stratified by HIV status. **Table S9.** Associations of GMB-associated metabolites with Plaque status, stratified by HIV status. **Table S10.** Correlations between bacterial species and Imidazole propionate. **Table S11.** Conditional analysis (mutual adjustment) highlight key ImP-associated GMB species out of ImP-correlated species. **Table S12.** Correlations between ImP associate bacterial species and *hutH. ***Table S13.** The presence of *hutH* in specific species: Sequence Alignment analyses.

## Data Availability

The gut microbiome sequence data in this study are deposited in QIITA database (https://qiita.ucsd.edu/) with accession number 13223. Data in this manuscript were collected by the Women’s Interagency HIV Study (WIHS), now the MACS/WIHS Combined Cohort Study (MWCCS). Access to individual-level data from the MACS/WIHS Combined Cohort Study Data (MWCCS) may be obtained upon review and approval of a MWCCS concept sheet. Links and instructions for online concept sheet submission are on the study website (https://statepi.jhsph.edu/mwccs/).
