# Supplementary material for: Gut microbiota, circulating inflammatory markers and metabolites, and carotid artery atherosclerosis in HIV infection
Source: Microbiome. 2023 May 27;11:119. doi: 10.1186/s40168-023-01566-2 (PMC10224225; doi:10.1186/s40168-023-01566-2)
Supplement: Supplementary file 2 — Additional file 1: Supplementary methods. Supplementary results. Figure S1. Number of participants for omics measurements. Figure S2. Microbial community level assessment. (A) α-diversity analyses by carotid artery plaque status. (B) Principal coordinates analysis (PCoA) of β-diversity using weighted UniFrac distances. Figure S3. Comparison of Amplicon 16S rRNA Sequencing and Shotgun Metagenomics Sequencing: plaque-associated bacterial genera. Figure S4. Spearman correlations between plaque-associated bacterial species and traditional CVD risk factors. Figure S5. Average relative abundance of plaque-associated species by viral suppression, among women on ART. Figure S6. Differentially abundant species according to Carotid Artery Plaque status, stratified by HIV infection. Figure S7. Correlations between plaque-associated bacterial species and plasma metabolites (A) polar metabolites (B) lipids. Figure S8. Associations between microbial species and plaque status, adjusted for metabolomic profiles. Table S1. Characteristics of study participants. Table S2. Characteristics of women on ART, by viral load. Table S3. Associations of gut microbial species with HIV status. Table S4. Associations of gut microbial species with HIV-specific variables, among women with HIV. Table S5. Associations of gut microbial species with Viral load, among women on ART. Table S6. Associations of bacterial species with Plaque status, stratified by HIV status. Table S7. GMB functional enzymes and carotid artery plaque: enrichment test. Table S8. Associations of proteomic inflammatory markers PLSDA PCs with plaque status, stratified by HIV status. Table S9. Associations of GMB-associated metabolites with Plaque status, stratified by HIV status. Table S10. Correlations between bacterial species and Imidazole propionate. Table S11. Conditional analysis (mutual adjustment) highlight key ImP-associated GMB species out of ImP-correlated species. Table S12. Correlations between ImP associate bacteri [file 40168_2023_1566_MOESM1_ESM.pdf]

Supplemental Materials

Supplemental Figures (Fig S1 – Fig S8)

Supplemental Tables (Table S1 – Table S13)

Supplemental Methods

Figure S1. Number of participants for omics measurements.

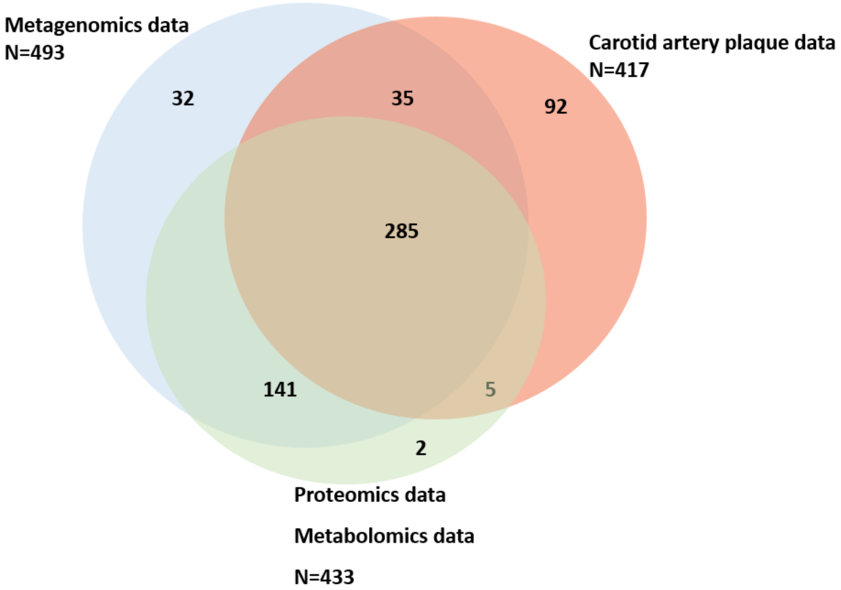

Figure S2. Microbial community level assessment

(A). α-diversity analyses by carotid artery plaque status. (all P > .05)

(B). Principal coordinates analysis (PCoA) of β-diversity using weighted UniFrac distances.

A

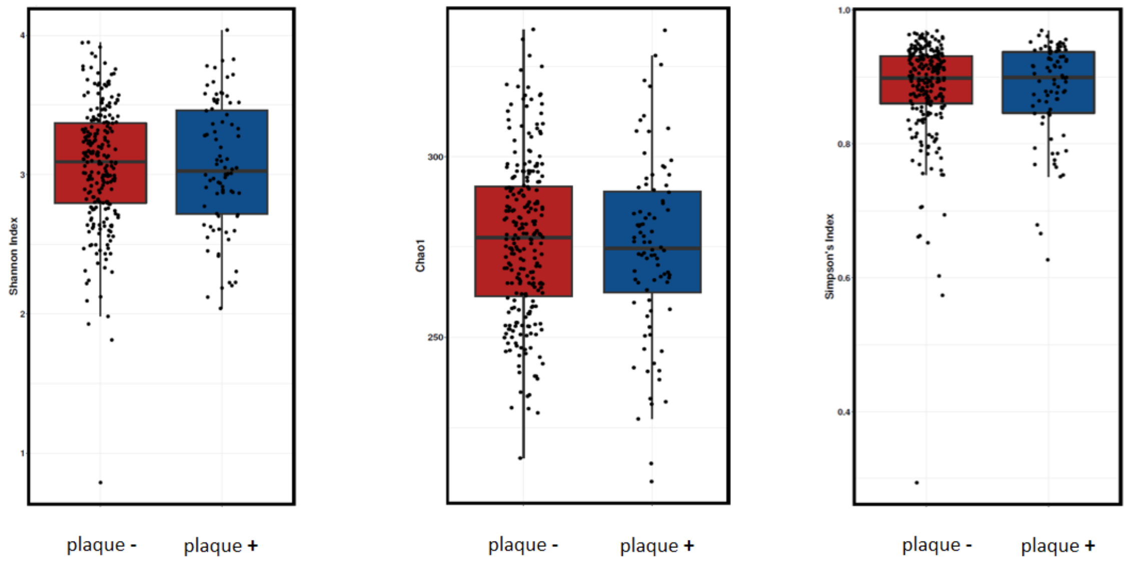

B

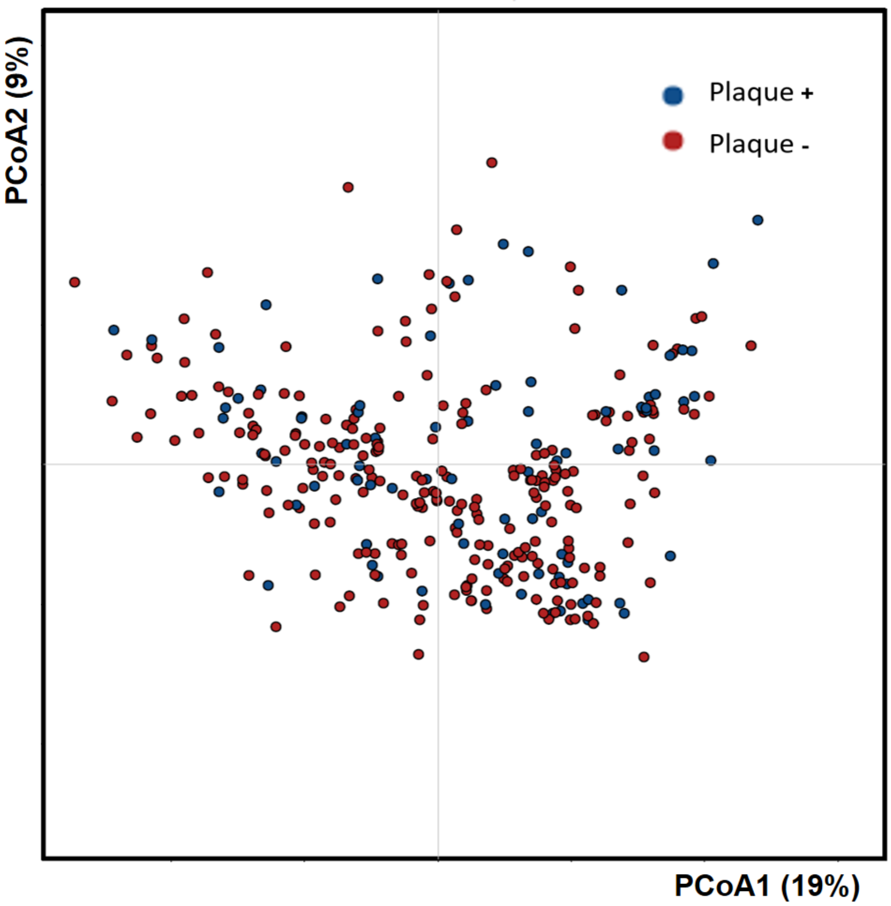

Figure S3. Comparison of Amplicon 16S rRNA Sequencing and Shotgun metagenomics Sequencing : plaque-associated bacterial genera.
Data are odds ratios (ORs) and 95% confidence intervals (CIs), adjusted for age, race, study site, antibiotics use, income, education, BMI, alcohol, smoking status, HIV status and ART use.

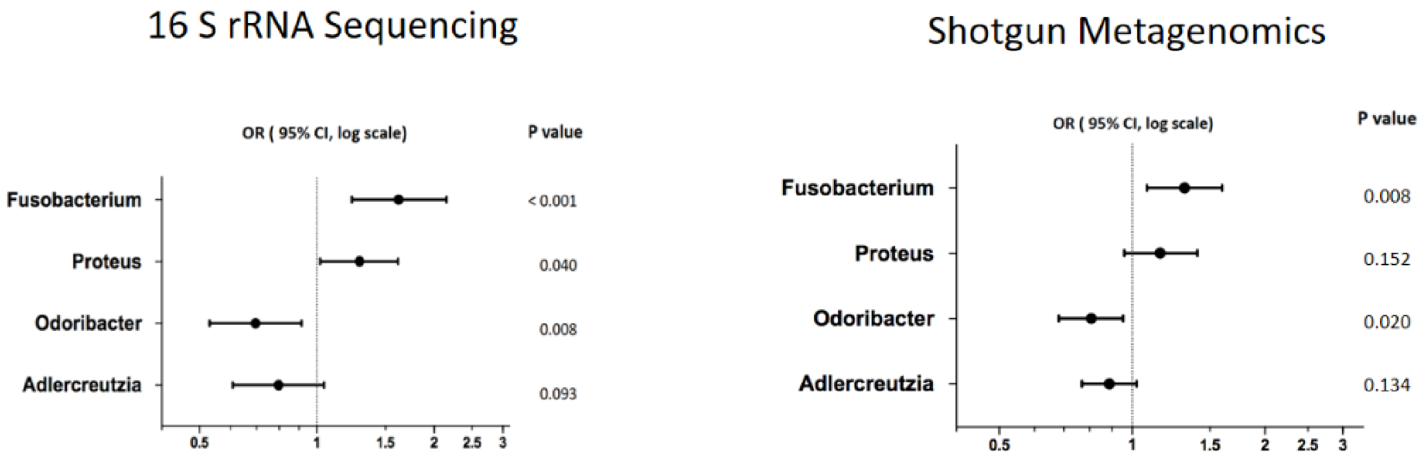

Figure S4. Spearman correlations between plaque-associated bacterial species and traditional CVD risk factors. *P < .05

Abbreviations: BMI, body mass index; SBP, Systolic blood pressure; DBP, Diastolic blood pressure;HDL, high-density lipoprotein; LDL, low-density lipoprotein; HbA1C, hemoglobin A1c.

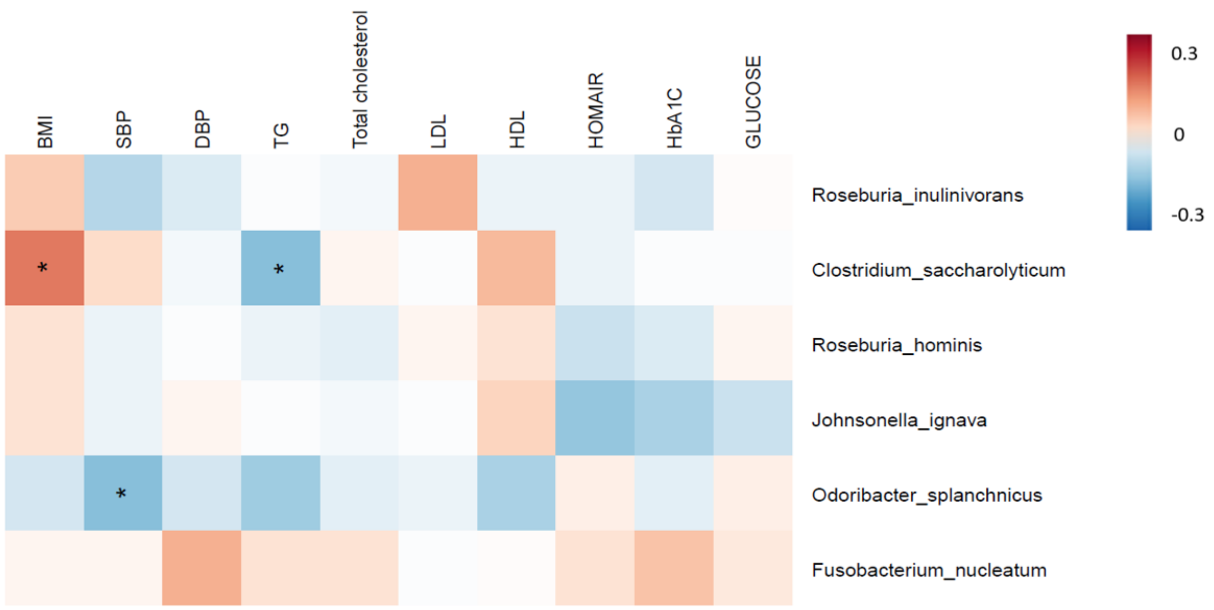

Figure S5. Average relative abundance of plaque-associated species by viral suppression, among women on ART.
Aviremic, undetectable HIV-1 viral load ≤20 copies/mL; Viremic, detectable HIV-1 viral load >20 copies/mL.

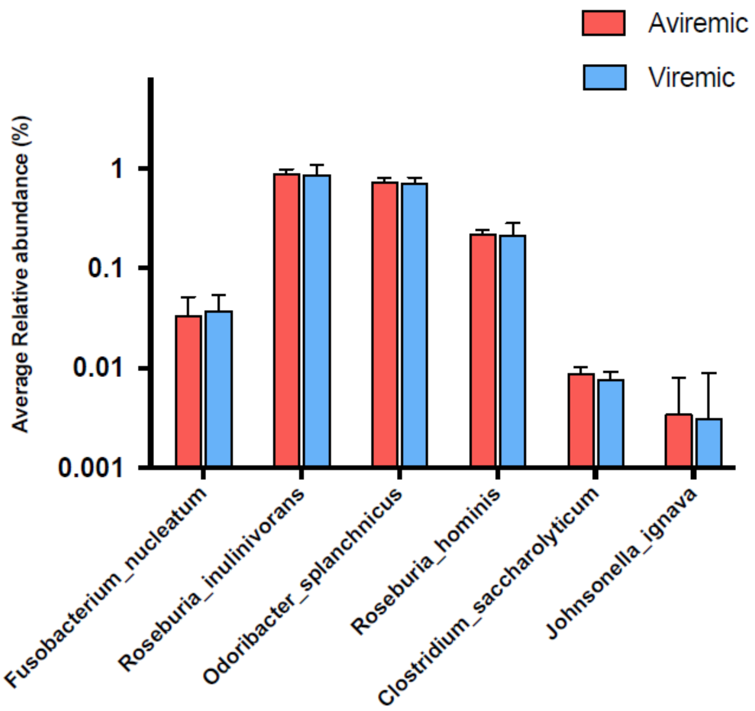

Figure S6. Differentially abundant species according to Carotid Artery Plaque status, stratified by HIV infection.

Taxonomic Linear discriminative analysis (LDA) effect size (LefSe) analysis by Carotid Artery Plaque status, stratified by HIV infection. (A) HIV positive samples (n=216); (B) HIV negative samples (n=104).

A

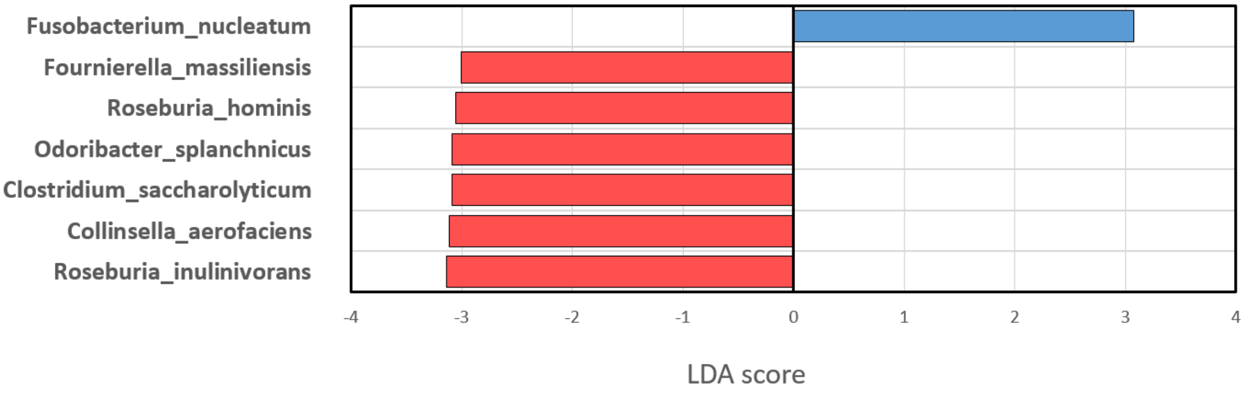

B

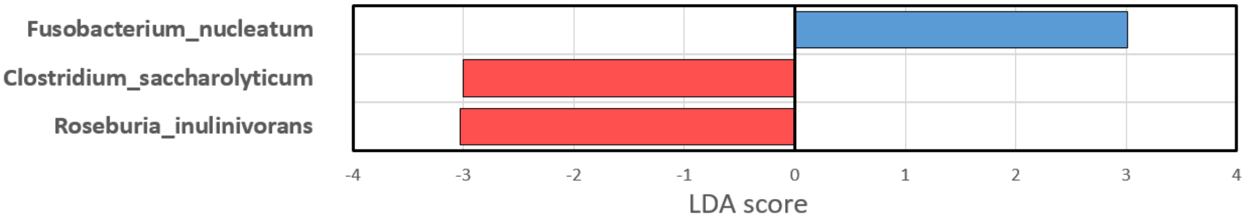

Figure S7. Correlations between plaque-associated bacterial species and plasma metabolites (FDR < 0.1)
(A) polar metabolites
(B) lipids

A

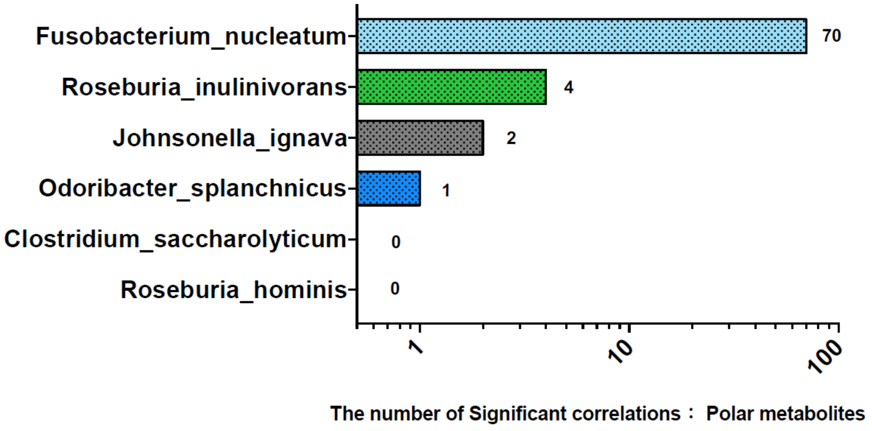

B

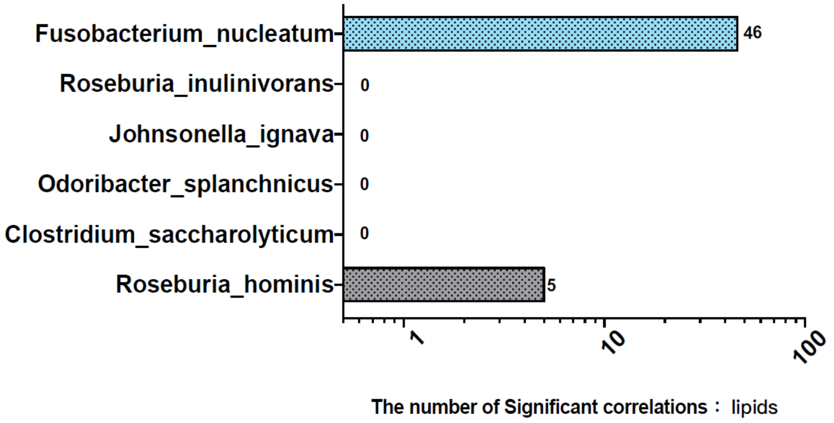

Figure S8. Associations between microbial species and plaque status, adjusted for metabolomic profiles.

Data are odds ratios (ORs) and 95% confidence intervals (CIs) for carotid artery plaque per standard deviation increment of CLR transformed abundance of gut bacterial species, adjusted for age, race, study site, antibiotics use, income, education, BMI, alcohol, smoking status, HIV status and ART use (model 1); further adjusted for 3-hydroxyhippuric acid (model 2).

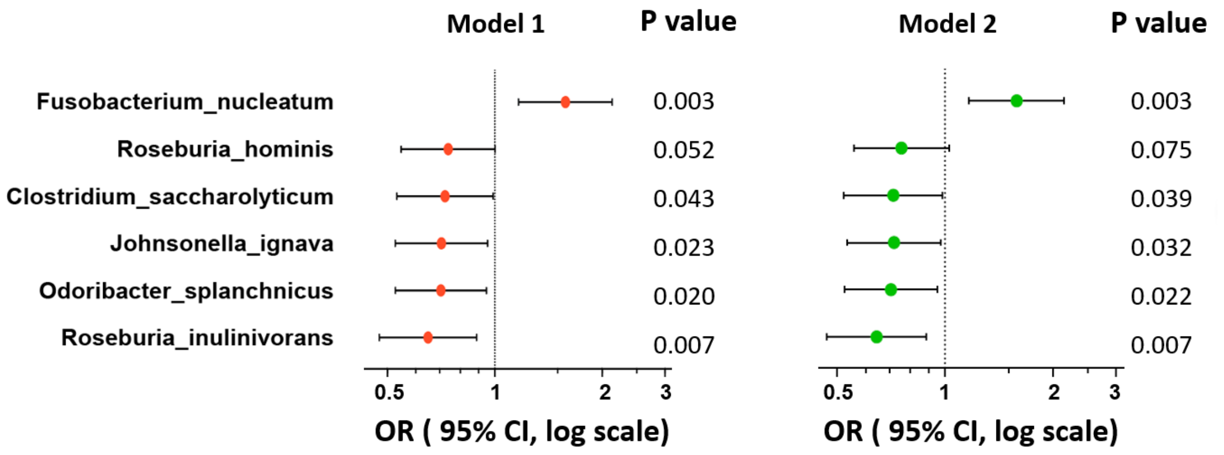

Table S1. Characteristics of study participants

	Carotid artery plaque -	Carotid artery Plaque +	P value
**Number of participants**	n=236	n=84	
**Age, y, median(IQR)**	51(46-56)	57 (53-62)	<0.001
**BMI, median(IQR)**	30.7 (26.1-36.4)	28.7 (25.4-33.87)	0.22
**Race, (%)**			0.51
African-American	61.5	67.9	
White	3.8	2.4	
Hispanic/Other	34.7	29.7	
**Annual Income** < $12,000, **(%)**	62.8	61.9	0.94
**Education** less than high school, **(%)**	46.6	42.9	0.59
**Smoking status , (%)**			0.03
Never smoker	23.5	14.3	
Current smoker	44.0	38.1	
Former smoker	32.5	47.6	
**Antibiotic use, (%)**	6.4	3.6	0.33
**Systolic blood pressure, mmHg**	126 (113 -141)	131 (118 -145)	0.06
**Diastolic blood pressure,mmHg**	77 (68-84)	78 (72-87)	0.08
**Triglycerides, mg/dL**	104 (76-146)	105 (77-162)	0.22
**Total cholesterol, mg/dL**	187 (161-213)	193 (160-215)	0.76
**HDL cholesterol, mg/dL**	56 (46-68)	57 (47-72)	0.22
**LDL cholesterol, mg/dL**	103 (82-126)	100 (82-128)	0.67
**Glucose, mg/dL**	84 (70-90)	84 (73-92)	0.68
**HbA1C , (%)**	5.5 (5.2-5.8)	5.5 (5.2-6.1)	0.48
**Anti-hypertensive medication, (%)**	39.6	55.8	0.02
**Anti-cholesterol medication, (%)**	20.3	28.6	0.14
**HIV-specific characteristics**			
**HIV positive, (%)**	64.5	67.9	0.76
**Detectable HIV-1 viral load, among HIV+, (%)**	27.1	23.7	0.61
**Low CD4 Count, among HIV+,(%)**	34.3	23.7	0.29
**ART use, among HIV+, (%)**	91.6	93.2	0.48

Data are presented as count (%) for categorical variables or median (IQR) for continuous variables unless otherwise noted.
Abbreviations: ART, antiretroviral therapy; HDL, high-density lipoprotein; HbA1C, hemoglobin A1c; HIV, human immunodeficiency virus; IQR, interquartile range; LDL, low-density lipoprotein.

Antibiotic use based on self-reported medication records at the time of closest core visit.

Table S2. Characteristics of women on ART , by viral load

	Detectable HIV-1 viral load	Non-Detectable HIV-1 viral load	P value
**Number of participants**	n=47	n=152	
**Age, y, median (IQR)**	53(48-58)	51 (49-55)	0.950
**BMI, median (IQR)**	28.1 (24.8-34.2)	31.2 (26.2-36.4)	0.015
**Race, (%)**			0.867
African American	68.1	61.3	
White	2.1	3.3	
Hispanic/Other	29.8	35.4	
**Annual Income** < $12,000, **(%)**	58.0	59.9	0.460
**Education** less than high school**, (%)**	44.7	48.6	0.574
**Smoking status, (%)**			0.187
Never smoker	17.0	27.3	
Current smoker	48.9	35.3	
Former smoker	34.0	37.3	
**Antibiotic use, (%)**	6.4	5.0	
**Systolic blood pressure, mmHg**	126(113 -138)	122 (115 -138)	0.658
**Diastolic blood pressure, mmHg**	78 (71-86)	76 (70-84)	0.940
**Triglycerides, mg/dL**	117 (88-158)	101 (67-141)	0.242
**Total cholesterol, mg/dL**	182 (142-218)	185 (154-214)	0.775
**HDL cholesterol, mg/dL**	51 (42-64)	56 (43-69)	0.327
**LDL cholesterol, mg/dL**	98 (71-140)	101 (75-125)	0.555
**Glucose, mg/dL**	89 (84-98)	89 (83-97)	0.546
**HbA1C**	5.6 (5.2-6.1)	5.6 (5.3-6.1)	0.401
**Anti-hypertensive medication, (%)**	36.2	40.7	0.854
**Anti-cholesterol medication, (%)**	10.6	22.0	0.227

Data are presented as count (%) for categorical variables or mean (SD) for continuous variables unless otherwise noted.
Abbreviations: ART, antiretroviral therapy; HDL, high-density lipoprotein; HbA1C, hemoglobin A1c; HIV, human immunodeficiency virus; IQR, interquartile range; LDL, low-density lipoprotein.

Antibiotic use based on self-reported medication records at the time of closest core visit.

Table S3. Associations of gut microbial species with HIV status (n= 320)

	HIV status (Ref: HIV -)		
Microbial species	Beta coefficient	SE	P
Fusobacterium_nucleatum	0.149	0.131	0.255
Odoribacter_splanchnicus	-0.143	0.132	0.281
Johnsonella_ignava	-0.081	0.131	0.539
Roseburia_hominis	-0.154	0.134	0.251
Clostridium_saccharolyticum	-0.090	0.131	0.495
Roseburia_inulinivorans	-0.012	0.132	0.926

Adjusted for age, race, study site, antibiotics use, income, education, BMI, alcohol, and smoking status.

Table S4. Associations of gut microbial species with HIV-specific variables, among women with HIV (n= 216)

	CD4 Count (Ref: high CD4 counts)			ART use (Ref: ART use group)			Viral load (Ref: undetectable HIV viral load)		
Microbial species	Beta	SE	P	Beta	SE	P	Beta	SE	P
Fusobacterium_nucleatum	0.038	0.175	0.827	-0.357	0.309	0.249	0.058	0.177	0.746
Odoribacter_splanchnicus	-0.097	0.171	0.570	-0.222	0.302	0.463	-0.056	0.179	0.756
Johnsonella_ignava	-0.205	0.160	0.202	0.089	0.291	0.761	0.008	0.173	0.964
Roseburia_hominis	-0.246	0.167	0.144	0.053	0.298	0.860	-0.036	0.177	0.838
Clostridium_saccharolyticum	-0.174	0.169	0.305	-0.059	0.300	0.844	-0.150	0.177	0.399
Roseburia_inulinivorans	-0.207	0.165	0.210	0.367	0.291	0.209	-0.017	0.173	0.921

Adjusted for age, race, study site, antibiotics use, income, education, BMI, alcohol, and smoking status.

High CD4 counts, ≥500 cells/mm^3; low CD4 counts ,<500 cells/mm^3.

Undetectable HIV viral load, ≤20 copies/mL; detectable HIV viral load, >20 copies/mL.

Table S5. Associations of gut microbial species with Viral load, among women on ART (n=199)

	Viral load		
Microbial species	Beta coefficient	SE	P
Fusobacterium_nucleatum	0.115	0.201	0.569
Odoribacter_splanchnicus	-0.002	0.198	0.999
Johnsonella_ignava	-0.069	0.192	0.720
Roseburia_hominis	-0.024	0.191	0.899
Clostridium_saccharolyticum	-0.202	0.197	0.307
Roseburia_inulinivorans	-0.153	0.189	0.421

Adjusted for age, race, study site, antibiotics use, income, education, BMI, alcohol, and smoking status.

Table S6. Associations of bacterial species with Plaque status, stratified by HIV status

species	Plaque HIV + (n= 216)				Plaque HIV - (n=104)				P for interaction
	OR	95CIlow	95CIup	P	OR	95CIlow	95CIup	P	
Roseburia_inulinivorans	0.67	0.52	0.87	0.003	0.67	0.45	0.98	0.040	0.836
Collinsella_aerofaciens	0.80	0.69	0.94	0.006	0.93	0.72	1.21	0.606	0.315
Fusobacterium_nucleatum	1.32	1.07	1.64	0.010	1.48	0.95	2.29	0.084	0.359
Odoribacter_splanchnicus	0.75	0.60	0.94	0.013	0.97	0.71	1.32	0.834	0.333
Fournierella_massiliensis	0.59	0.37	0.93	0.023	0.82	0.41	1.65	0.581	0.372
Roseburia_hominis	0.70	0.51	0.97	0.034	0.92	0.54	1.56	0.747	0.336
Clostridium_saccharolyticum	0.75	0.57	0.99	0.043	0.69	0.49	0.96	0.027	0.837
Johnsonella_ignava	0.74	0.55	1.01	0.061	0.98	0.60	1.60	0.942	0.382

Adjusted for age, race, study site, antibiotics use, income, education, BMI, alcohol, and smoking status.

Table S7. GMB functional enzymes and carotid artery plaque: enrichment test

Enzyme LV3	Significant Enzymes	Not Significant	Total Enzymes of the category	Enrichment test p value
6.3.4 Carbon-nitrogen ligases	5	6	11	**0.002** *
1.3.1 With NAD+ or NADP+ as acceptor	4	8	12	**0.027**
3.1.1 Carboxylic-ester hydrolases	4	10	14	**0.046**
2.1.1 Methyltransferases	12	63	75	0.077
3.1.3 Phosphoric-monoester hydrolases	6	27	33	0.112
6.3.2 Acid-D-amino-acid ligases (peptide synthases)	3	11	14	0.162
6.1.1 Ligases forming aminoacyl-tRNA and related compounds	5	24	29	0.165
2.7.13 Protein-histidine kinases	5	25	30	0.182
3.6.1 In phosphorus-containing anhydrides	4	19	23	0.198
3.6.4 Acting on acid anhydrides to facilitate cellular and subcellular movement	3	13	16	0.215
5.2.1 cis-trans Isomerases (only sub-subclass identified to date)	2	9	11	0.307
6.3.5 Carbon-nitrogen ligases with glutamine as amido-N-donor	2	9	11	0.307
2.5.1 Transferring alkyl or aryl groups, other than methyl groups	5	32	37	0.317
2.7.7 Nucleotidyltransferases	7	52	59	0.386
3.5.4 In cyclic amidines	2	12	14	0.420
2.7.8 Transferases for other substituted phosphate groups	2	14	16	0.489
2.6.1 Transaminases	3	23	26	0.495
2.4.2 Pentosyltransferases	3	24	27	0.521
2.7.1 Phosphotransferases with an alcohol group as acceptor	8	70	78	0.533
1.2.1 With NAD+ or NADP+ as acceptor	2	16	18	0.554
2.3.1 Transferring groups other than aminoacyl groups	5	56	61	0.745
4.1.3 Oxo-acid-lyases	1	12	13	0.748
1.1.1 With NAD+ or NADP+ as acceptor	6	71	77	0.795
4.1.2 Aldehyde-lyases	1	14	15	0.796
5.1.3 Acting on carbohydrates and derivatives	1	16	17	0.835
3.6.3 Acting on acid anhydrides	3	42	45	0.842
5.4.99 Transferring other groups	1	18	19	0.866
5.3.1 Interconverting aldoses and ketoses, and related compounds	1	19	20	0.879
2.4.1 Hexosyltransferases	3	47	50	0.888
3.5.1 In linear amides	1	28	29	0.953
4.1.1 Carboxy-lyases	1	34	35	0.975
3.2.1 Glycosidases, i.e. enzymes that hydrolyse O- and S-glycosyl compounds	1	57	58	0.998
4.2.1 Hydro-lyases	0	55	55	0.999
1.6.5 With a quinone or similar compound as acceptor	0	23	23	0.999
1.2.7 With an iron-sulfur protein as acceptor	0	14	14	0.999
3.2.2 Hydrolysing N-glycosyl compounds	0	11	11	0.999
5.4.2 Phosphotransferases (phosphomutases)	0	11	11	0.999
1.3.99 With other acceptors	0	10	10	0.999
2.7.4 Phosphotransferases with a phosphate group as acceptor	0	10	10	0.999
2.8.3 CoA-transferases	0	10	10	0.999
6.2.1 Acid-thiol ligases	0	10	10	0.999

* FDR <0.10

Table S8. Associations of proteomic inflammatory markers PLSDA PCs with plaque status, stratified by HIV status

	All individuals (n= 290)				HIV + (n= 198)				HIV - (n=92)				P for interaction
PLSDA PCs	OR	95CIlow	95CIup	P	OR	95CIlow	95CIup	P	OR	95CIlow	95CIup	P	
PC1	1.24	1.09	1.41	<0.001*	1.23	1.06	1.43	0.007	1.38	1.04	1.84	0.025	0.670
PC2	1.29	1.11	1.50	0.002*	1.27	1.07	1.53	0.008	1.28	0.94	1.75	0.113	0.840
PC3	1.49	1.20	1.85	<0.001*	1.57	1.22	2.02	<0.001	1.32	0.83	2.10	0.241	0.331
PC4	2.07	1.53	2.79	<0.001*	2.21	1.55	3.14	<0.001	1.90	1.00	3.61	0.050	0.644
PC5	1.53	1.13	2.06	0.006*	1.55	1.10	2.20	0.013	1.84	0.94	3.61	0.077	0.705
PC6	1.11	0.95	1.25	0.14	1.04	0.90	1.20	0.566	1.36	0.95	1.96	0.094	0.233
PC7	1.16	0.94	1.43	0.231	1.18	0.92	1.51	0.184	1.46	0.80	2.65	0.214	0.523
PC8	1.08	0.89	1.28	0.38	1.06	0.85	1.31	0.609	1.08	0.75	1.57	0.668	0.996

Adjusted for age, race, study site, income, education, BMI, alcohol, and smoking status; (and HIV status in non-stratified analyses) .
*FDR P<0.05

Table S9. Associations of GMB-associated metabolites with Plaque status, stratified by HIV status

	HIV + (n= 198)				HIV - (n=92)				P for interaction
Metabolites	OR	95CIlow	95CIup	P	OR	95CIlow	95CIup	P	
Imidazole propionate	1.35	0.83	2.19	0.231	1.39	0.70	2.75	0.351	0.917
3-Hydroxyhippuric acid	1.40	1.00	1.95	0.051	1.38	0.85	2.25	0.196	0.868
Ribothymidine	1.16	0.76	1.77	0.494	0.91	0.55	1.50	0.704	0.586
L-Urobilin	0.73	0.40	1.36	0.323	1.12	0.63	1.97	0.704	0.379
Ornithine	0.94	0.62	1.42	0.756	0.77	0.44	1.35	0.355	0.625

Adjusted for age, race, study site, income, education, BMI, alcohol, and smoking status.

Table S10. Correlations between bacterial species and Imidazole propionate

species	Spearman Correlation coefficient	P value	FDR P
Ruminococcus_gnavus	0.332	1.04E-08	1.72E-06
Clostridium_bolteae	0.319	4.08E-08	3.38E-06
Coprococcus_sp._HPP0048	0.309	1.15E-07	4.85E-06
Erysipelatoclostridium_ramosum	0.309	1.17E-07	4.85E-06
Eggerthella_lenta	0.302	2.26E-07	8.34E-06
Flavonifractor_Flavonifractor_plautii	0.292	5.55E-07	1.67E-05
Lachnospiraceae_bacterium_2_1_46FAA	0.287	8.77E-07	2.43E-05
Citrobacter_Citrobacter_freundii	0.282	1.37E-06	3.24E-05
Anaerostipes_Anaerostipes_caccae	0.273	3.16E-06	6.16E-05
Sellimonas_Sellimonas_intestinalis	0.238	5.33E-05	7.07E-04
Lachnoclostridium_Clostridium_symbiosum	0.227	1.20E-04	1.37E-03
Blautia_Blautia_producta	0.224	1.46E-04	1.61E-03
Lachnoclostridium_Clostridium_scindens	0.223	1.50E-04	1.61E-03
Escherichia_Escherichia_coli	0.223	1.57E-04	1.63E-03
Klebsiella_Klebsiella_aerogenes	0.222	1.65E-04	1.66E-03
Veillonella_Veillonella_parvula	0.221	1.74E-04	1.70E-03
Enterococcus_Enterococcus_avium	0.205	5.08E-04	4.02E-03
Shigella_Shigella_dysenteriae	0.203	5.84E-04	4.51E-03
Erysipelatoclostridium_Clostridium_innocuum	0.197	8.48E-04	5.92E-03
Shigella_Shigella_flexneri	0.197	8.56E-04	5.92E-03
Streptococcus_Streptococcus_anginosus	0.195	9.60E-04	6.25E-03
Streptococcus_Streptococcus_gallolyticus	0.194	1.05E-03	6.58E-03
Lachnoclostridium_Clostridium_glycyrrhizinilyticum	0.191	1.22E-03	7.38E-03
Enterococcus_Enterococcus_faecalis	0.189	1.37E-03	7.97E-03
Blautia_Blautia_hansenii	0.179	2.56E-03	1.37E-02
Lawsonella_Lawsonella_clevelandensis	0.178	2.72E-03	1.43E-02
Streptococcus_Streptococcus_mitis	0.171	3.88E-03	1.83E-02
Streptococcus_Streptococcus_sanguinis	0.171	3.92E-03	1.83E-02
Enterobacter_Enterobacter_cloacae	0.170	4.22E-03	1.94E-02
Klebsiella_Klebsiella_oxytoca	0.168	4.58E-03	2.03E-02
Morganella_Morganella_morganii	0.167	4.83E-03	2.11E-02
Trueperella_Trueperella_pyogenes	0.165	5.32E-03	2.24E-02
Streptococcus_Streptococcus_parasanguinis	0.161	6.71E-03	2.69E-02
Leclercia_Leclercia_adecarboxylata	0.156	8.49E-03	3.17E-02
Enterococcus_Enterococcus_faecium	0.154	9.58E-03	3.53E-02
Cronobacter_Cronobacter_sakazakii	0.150	1.15E-02	4.01E-02
Haemophilus_Haemophilus_sputorum	0.149	1.21E-02	4.11E-02
Fusobacterium_Fusobacterium_mortiferum	0.148	1.27E-02	4.23E-02
Lactobacillus_Lactobacillus_vaginalis	0.146	1.40E-02	4.55E-02
Lactobacillus_Lactobacillus_antri	0.146	1.41E-02	4.55E-02
Campylobacter_Campylobacter_gracilis	0.146	1.41E-02	4.55E-02
Moraxella_Moraxella_osloensis	0.142	1.65E-02	5.17E-02
Kluyvera_Kluyvera_cryocrescens	0.142	1.65E-02	5.17E-02
Lactobacillus_Lactobacillus_rhamnosus	0.142	1.67E-02	5.17E-02
Peptoniphilus_Peptoniphilus_lacrimalis	0.140	1.83E-02	5.56E-02
Streptococcus_Streptococcus_gordonii	0.138	1.99E-02	5.89E-02
Streptococcus_Streptococcus_oralis	0.136	2.19E-02	6.39E-02
Parvimonas_Parvimonas_micra	0.136	2.25E-02	6.44E-02
Megasphaera_Megasphaera_sp._BV3C161	0.132	2.69E-02	7.57E-02
Lachnoclostridium_Clostridium_citroniae	0.131	2.72E-02	7.58E-02
Lactobacillus_Lactobacillus_mucosae	0.131	2.75E-02	7.58E-02
Proteus_Proteus_mirabilis	0.131	2.76E-02	7.58E-02
Klebsiella_Klebsiella_pneumoniae	0.131	2.79E-02	7.60E-02
Lactobacillus_Lactobacillus_fermentum	0.129	3.02E-02	7.87E-02

Bifidobacterium_Bifidobacterium_reuteri	0.129	3.03E-02	7.87E-02
Pseudomonas_Pseudomonas_otitidis	0.127	3.32E-02	8.49E-02
Dysgonomonas_Dysgonomonas_capnocytophagoides	0.127	3.33E-02	8.49E-02
Citrobacter_Citrobacter_amalonaticus	0.125	3.54E-02	8.84E-02
Clostridium_Clostridium_ventriculi	0.123	3.84E-02	9.36E-02
Pediococcus_Pediococcus_acidilactici	0.122	3.96E-02	9.60E-02
Intestinibacter_Intestinibacter_bartlettii	0.122	4.06E-02	9.73E-02
Pontibacillus_Pontibacillus_chungwhensis	0.121	4.12E-02	9.73E-02
Faecalicatena_Faecalicatena_fissicatena	0.121	4.12E-02	9.73E-02
Actinomyces_Actinomyces_oris	0.121	4.13E-02	9.73E-02
Streptococcus_Streptococcus_infantis	0.120	4.28E-02	9.89E-02
Bittarella_Bittarella_massiliensis	-0.120	4.29E-02	9.89E-02
Prevotella_Prevotella_sp._HUN102	-0.121	4.22E-02	9.86E-02
Paraprevotella_Paraprevotella_xylaniphila	-0.124	3.66E-02	9.00E-02
Prevotella_Prevotella_bergensis	-0.125	3.59E-02	8.89E-02
Prevotella_Prevotella_bryantii	-0.125	3.53E-02	8.84E-02
Prevotella_Prevotella_sp._P476	-0.126	3.35E-02	8.49E-02
Alistipes_Alistipes_ihumii	-0.129	2.95E-02	7.77E-02
Porphyromonas_Porphyromonas_crevioricanis	-0.130	2.89E-02	7.68E-02
Fournierella_Fournierella_massiliensis	-0.130	2.86E-02	7.65E-02
Butyricimonas_Butyricimonas_synergistica	-0.130	2.83E-02	7.63E-02
Roseburia_Roseburia_intestinalis	-0.135	2.34E-02	6.63E-02
Bacteria_bacterium_LF3	-0.136	2.23E-02	6.44E-02
Eubacterium_Eubacterium_ventriosum	-0.136	2.18E-02	6.39E-02
Marvinbryantia_Marvinbryantia_formatexigens	-0.139	1.90E-02	5.69E-02
Prevotella_Prevotella_stercorea	-0.140	1.85E-02	5.60E-02
Butyricimonas_Butyricimonas_virosa	-0.145	1.43E-02	4.56E-02
Eubacterium_Eubacterium_eligens	-0.148	1.27E-02	4.23E-02
Coprobacter_Coprobacter_secundus	-0.150	1.18E-02	4.03E-02
Anaerotruncus_Anaerotruncus_sp._G32012	-0.150	1.16E-02	4.02E-02
Streptococcus_Streptococcus_agalactiae	-0.153	9.87E-03	3.56E-02
Eubacterium_Eubacterium_hallii	-0.153	9.87E-03	3.56E-02
Kandleria_Kandleria_vitulina	-0.156	8.42E-03	3.17E-02
Dorea_Dorea_longicatena	-0.158	7.77E-03	2.96E-02
Clostridioides_Clostridioides_difficile	-0.159	7.52E-03	2.90E-02
Kluyvera_Kluyvera_ascorbata	-0.160	6.98E-03	2.73E-02
Holdemania_Holdemania_massiliensis	-0.161	6.62E-03	2.68E-02
Rikenella_Rikenella_microfusus	-0.163	6.10E-03	2.53E-02
Ruminococcus_Ruminococcus_faecis	-0.166	5.04E-03	2.14E-02
Senegalimassilia_Senegalimassilia_anaerobia	-0.166	5.00E-03	2.14E-02
Roseburia_hominis	-0.168	4.51E-03	2.02E-02
Clostridium_saccha	-0.169	4.40E-03	2.00E-02
Cellulomonas_Cellulomonas_carbonis	-0.172	3.74E-03	1.80E-02
Odoribacter_splanc	-0.172	3.72E-03	1.80E-02
Lachnospiraceae_Lachnospiraceae_bacterium_COE1	-0.173	3.53E-03	1.75E-02
Catenibacterium_Catenibacterium_mitsuokai	-0.175	3.18E-03	1.60E-02
Alistipes_Alistipes_indistinctus	-0.175	3.17E-03	1.60E-02
Collinsella_Collinsella_sp._MS5	-0.175	3.06E-03	1.59E-02
Prevotella_Prevotella_copri	-0.179	2.46E-03	1.34E-02

Subdoligranulum_Subdoligranulum_variabile	-0.184	1.91E-03	1.06E-02
Coprococcus_Coprococcus_eutactus	-0.187	1.58E-03	8.92E-03
Enterococcus_Enterococcus_gilvus	-0.187	1.54E-03	8.84E-03
Lachnospiraceae_Lachnospiraceae_bacterium_TF0111	-0.191	1.27E-03	7.54E-03
Adlercreutzia_Adlercreutzia_equolifaciens	-0.193	1.07E-03	6.60E-03
Clostridiales_Bacteroides_pectinophilus	-0.194	1.03E-03	6.58E-03
Lachnoanaerobaculum_Lachnoanaerobaculum_sp._OBRC55	-0.196	9.29E-04	6.17E-03
Pseudoflavonifractor_Pseudoflavonifractor_capillosus	-0.197	8.74E-04	5.92E-03
Alistipes_Alistipes_inops	-0.199	7.48E-04	5.40E-03
Barnesiella_Barnesiella_intestinihominis	-0.200	7.36E-04	5.40E-03
Oscillibacter_Oscillibacter_sp._13	-0.201	6.67E-04	5.03E-03
Alistipes_Alistipes_finegoldii	-0.207	4.53E-04	3.66E-03
Roseburia_inuliniv	-0.210	3.73E-04	3.10E-03
Alistipes_Alistipes_senegalensis	-0.210	3.67E-04	3.10E-03
Lachnospiraceae_Lachnospiraceae_bacterium_oral_taxon_50(	-0.212	3.38E-04	2.95E-03
Holdemanella_Holdemanella_biformis	-0.214	2.96E-04	2.66E-03
Alistipes_Alistipes_timonensis	-0.214	2.83E-04	2.61E-03
Ruminococcus_Ruminococcus_bicirculans	-0.218	2.16E-04	2.04E-03
Coprococcus_Coprococcus_comes	-0.228	1.10E-04	1.30E-03
Alistipes_Alistipes_obesi	-0.233	7.82E-05	9.61E-04
Faecalicoccus_Faecalicoccus_pleomorphus	-0.234	6.95E-05	8.88E-04
Johnsonella_ignava	-0.240	4.46E-05	6.26E-04
Faecalibacterium_Faecalibacterium_prausnitzii	-0.246	2.95E-05	4.45E-04
Clostridium_Clostridium_phoceensis	-0.255	1.39E-05	2.20E-04
Dorea_Dorea_formicigenerans	-0.259	1.06E-05	1.76E-04
Alistipes_Alistipes_putredinis	-0.268	4.72E-06	8.25E-05
Alistipes_Alistipes_shahii	-0.272	3.34E-06	6.16E-05
Intestinimonas_Intestinimonas_massiliensis	-0.272	3.31E-06	6.16E-05
Ruminiclostridium_Eubacterium_siraeum	-0.280	1.76E-06	3.88E-05
Ruminococcus_Ruminococcus_callidus	-0.285	1.05E-06	2.68E-05
Butyrivibrio_Butyrivibrio_crossotus	-0.300	2.82E-07	9.38E-06
Clostridia_Clostridia_bacterium_UC5.11E11	-0.309	1.15E-07	4.85E-06
Intestinimonas_Intestinimonas_butyriciproducens	-0.316	5.67E-08	3.76E-06
Ruminococcus_Ruminococcus_champanellensis	-0.323	2.80E-08	3.10E-06
Oscillibacter_Oscillibacter_sp._ER4	-0.343	3.25E-09	1.08E-06

Table S11. Conditional analysis (mutual adjustment) highlight key ImP-associated GMB species out of ImP-correlated species

GMB Species	Beta	SE	P
Blautia_Blautia_hansenii	0.282	0.070	<0.001
Lachnoanaerobaculum_Lachnoanaerobaculum_sp._OBRC55	-0.273	0.080	<0.001
Dysgonomonas_Dysgonomonas_capnocytophagoides	0.210	0.075	0.006
Bifidobacterium_Bifidobacterium_reuteri	0.101	0.043	0.021
Clostridioides_Clostridioides_difficile	-0.160	0.071	0.025
Coprobacter_Coprobacter_secundus	-0.142	0.065	0.031
Pseudoflavonifractor_Pseudoflavonifractor_capillosus	-0.222	0.107	0.039
Fournierella_Fournierella_massiliensis	-0.220	0.107	0.042
Megasphaera_Megasphaera_sp._BV3C161	0.091	0.045	0.048
Lachnoclostridium_Clostridium_scindens	0.112	0.057	0.049
Kluyvera_Kluyvera_ascorbata	-0.159	0.081	0.051
Streptococcus_Streptococcus_agalactiae	-0.083	0.042	0.052
Pediococcus_Pediococcus_acidilactici	0.112	0.060	0.063
Roseburia_hominis	-0.160	0.087	0.067
Veillonella_Veillonella_parvula	0.073	0.043	0.088
Citrobacter_Citrobacter_freundii	0.083	0.049	0.089
Paraprevotella_Paraprevotella_xylaniphila	-0.078	0.046	0.092
Coprococcus_Coprococcus_sp._HPP0048	-0.082	0.051	0.107
Morganella_Morganella_morganii	-0.086	0.055	0.120
Actinomyces_Actinomyces_oris	-0.082	0.053	0.121
Enterococcus_Enterococcus_faecalis	0.056	0.038	0.144
Klebsiella_Klebsiella_oxytoca	0.075	0.052	0.148
Erysipelatoclostridium_Clostridium_innocuum	0.100	0.069	0.148
Lactobacillus_Lactobacillus_rhamnosus	-0.072	0.050	0.150
Proteus_Proteus_mirabilis	0.062	0.043	0.152
Enterococcus_Enterococcus_gilvus	-0.062	0.043	0.153
Prevotella_Prevotella_bergensis	0.060	0.043	0.166
Streptococcus_Streptococcus_sanguinis	0.105	0.076	0.168
Holdemania_Holdemania_massiliensis	-0.090	0.066	0.172
Lawsonella_Lawsonella_clevelandensis	0.087	0.063	0.173
Eggerthella_Eggerthella_lenta	0.072	0.053	0.177
Faecalicatena_Faecalicatena_fissicatena	0.062	0.047	0.189
Faecalicoccus_Faecalicoccus_pleomorphus	-0.094	0.074	0.204
Enterobacter_Enterobacter_cloacae	0.060	0.048	0.213
Blautia_Blautia_producta	0.075	0.061	0.220
Porphyromonas_Porphyromonas_crevioricanis	0.060	0.050	0.232
Roseburia_Roseburia_intestinalis	-0.063	0.053	0.238
Parvimonas_Parvimonas_micra	-0.063	0.054	0.244
Alistipes_Alistipes_ihumii	0.033	0.029	0.247
Subdoligranulum_Subdoligranulum_variabile	-0.120	0.104	0.252
Oscillibacter_Oscillibacter_sp._13	-0.109	0.096	0.260
Prevotella_Prevotella_sp._HUN102	0.051	0.045	0.261
Pseudomonas_Pseudomonas_otitidis	-0.081	0.075	0.281
Lachnoclostridium_Clostridium_bolteae	-0.097	0.094	0.300
Clostridiales_Bacteroides_pectinophilus	-0.112	0.109	0.304
Faecalibacterium_Faecalibacterium_prausnitzii	0.061	0.061	0.315
Streptococcus_Streptococcus_gallolyticus	0.037	0.037	0.324
Anaerostipes_Anaerostipes_caccae	0.042	0.043	0.325

Holdemanella_Holdemanella_biformis	0.045	0.045	0.326
Haemophilus_Haemophilus_sputorum	-0.048	0.049	0.331
Shigella_Shigella_flexneri	0.037	0.038	0.332
Senegalimassilia_Senegalimassilia_anaerobia	0.044	0.046	0.344
Flavonifractor_Flavonifractor_plautii	0.063	0.067	0.347
Escherichia_Escherichia_coli	-0.049	0.053	0.364
Intestinimonas_Intestinimonas_massiliensis	0.072	0.079	0.365
Clostridia_Clostridia_bacterium_UC5.11E11	0.071	0.078	0.367
Lachnospiraceae_Lachnospiraceae_bacterium_TF0111	0.083	0.092	0.369
Ruminococcus_gnavus	-0.062	0.070	0.378
Alistipes_Alistipes_senegalensis	0.053	0.060	0.378
Campylobacter_Campylobacter_gracilis	-0.055	0.063	0.389
Alistipes_Alistipes_indistinctus	0.022	0.025	0.395
Pontibacillus_Pontibacillus_chungwhensis	0.065	0.078	0.402
Lactobacillus_Lactobacillus_fermentum	-0.042	0.050	0.404
Cronobacter_Cronobacter_sakazakii	0.066	0.079	0.405
Intestinimonas_Intestinimonas_butyriciproducens	-0.068	0.085	0.420
Butyrivibrio_Butyrivibrio_crossotus	-0.059	0.073	0.421
Ruminococcus_Ruminococcus_callidus	0.038	0.047	0.423
Marvinbryantia_Marvinbryantia_formatexigens	-0.061	0.078	0.432
Lachnospiraceae_Lachnospiraceae_bacterium_oral_taxon_500	0.044	0.057	0.437
Lactobacillus_Lactobacillus_vaginalis	0.046	0.060	0.443
Ruminococcus_Ruminococcus_bicirculans	-0.027	0.036	0.446
Lachnospiraceae_Lachnospiraceae_bacterium_2_1_46FAA	0.061	0.085	0.479
Anaerotruncus_Anaerotruncus_sp._G32012	-0.071	0.101	0.484
Lachnoclostridium_Clostridium_citroniae	0.069	0.099	0.485
Moraxella_Moraxella_osloensis	-0.053	0.078	0.494
Lachnospiraceae_Lachnospiraceae_bacterium_COE1	0.045	0.065	0.496
Prevotella_Prevotella_copri	0.023	0.034	0.501
Leclercia_Leclercia_adecarboxylata	-0.060	0.097	0.537
Johnsonella_ignava	0.047	0.077	0.540
Erysipelatoclostridium_Erysipelatoclostridium_ramosum	-0.023	0.039	0.544
Lachnoclostridium_Clostridium_symbiosum	0.047	0.077	0.544
Oscillibacter_Oscillibacter_sp._ER4	0.042	0.071	0.558
Roseburia_inuliniv	0.042	0.072	0.559
Streptococcus_Streptococcus_anginosus	0.028	0.049	0.561
Alistipes_Alistipes_finegoldii	-0.026	0.050	0.598
Eubacterium_Eubacterium_eligens	0.023	0.044	0.598
Barnesiella_Barnesiella_intestinihominis	0.016	0.031	0.601
Clostridium_Clostridium_phoceensis	0.037	0.071	0.605
Clostridium_Clostridium_ventriculi	0.033	0.065	0.610
Peptoniphilus_Peptoniphilus_lacrimalis	-0.032	0.063	0.616
Dorea_Dorea_formicigenerans	-0.037	0.077	0.635
Streptococcus_Streptococcus_oralis	-0.029	0.062	0.636
Prevotella_Prevotella_bryantii	-0.020	0.042	0.638
Klebsiella_Klebsiella_pneumoniae	0.016	0.036	0.650
Alistipes_Alistipes_putredinis	-0.018	0.040	0.659
Prevotella_Prevotella_stercorea	0.019	0.045	0.675
Kandleria_Kandleria_vitulina	-0.023	0.056	0.676
Coprococcus_Coprococcus_comes	0.025	0.061	0.677
Streptococcus_Streptococcus_mitis	0.025	0.063	0.692
Catenibacterium_Catenibacterium_mitsuokai	-0.016	0.042	0.700
Rikenella_Rikenella_microfusus	-0.016	0.041	0.703
Trueperella_Trueperella_pyogenes	-0.025	0.066	0.708

Enterococcus_Enterococcus_avium	-0.020	0.055	0.720
Alistipes_Alistipes_obesi	-0.014	0.040	0.723
Streptococcus_Streptococcus_infantis	-0.023	0.070	0.741
Adlercreutzia_Adlercreutzia_equolifaciens	0.010	0.030	0.750
Fusobacterium_Fusobacterium_mortiferum	-0.011	0.034	0.752
Cellulomonas_Cellulomonas_carbonis	-0.016	0.050	0.754
Enterococcus_Enterococcus_faecium	-0.012	0.037	0.755
Kluyvera_Kluyvera_cryocrescens	-0.020	0.065	0.759
Dorea_Dorea_longicatena	0.015	0.052	0.768
Citrobacter_Citrobacter_amalonaticus	0.015	0.057	0.791
Collinsella_Collinsella_sp._MS5	-0.013	0.054	0.808
Lactobacillus_Lactobacillus_mucosae	-0.012	0.052	0.810
Bacteria_bacterium_LF3	0.010	0.042	0.822
Lachnoclostridium_Clostridium_glycyrrhizinilyticum	-0.019	0.085	0.826
Ruminococcus_Ruminococcus_faecis	0.011	0.055	0.836
Alistipes_Alistipes_timonensis	-0.009	0.045	0.844
Butyricimonas_Butyricimonas_synergistica	-0.007	0.044	0.873
Prevotella_Prevotella_sp._P476	0.007	0.045	0.882
Alistipes_Alistipes_inops	0.006	0.041	0.887
Streptococcus_Streptococcus_parasanguinis	0.010	0.072	0.894
Clostridium_saccha	0.005	0.059	0.929
Ruminiclostridium_Eubacterium_siraeum	0.003	0.033	0.931
Shigella_Shigella_dysenteriae	0.004	0.045	0.931
Alistipes_Alistipes_shahii	-0.005	0.054	0.931
Odoribacter_splanc	-0.004	0.050	0.942
Coprococcus_Coprococcus_eutactus	-0.004	0.060	0.949
Eubacterium_Eubacterium_ventriosum	0.004	0.068	0.956
Sellimonas_Sellimonas_intestinalis	0.002	0.045	0.959
Lactobacillus_Lactobacillus_antri	0.004	0.076	0.960
Butyricimonas_Butyricimonas_virosa	-0.002	0.038	0.962
Ruminococcus_Ruminococcus_champanellensis	-0.002	0.054	0.966
Eubacterium_Eubacterium_hallii	-0.002	0.052	0.967
Streptococcus_Streptococcus_gordonii	-0.002	0.066	0.975
Klebsiella_Klebsiella_aerogenes	0.000	0.052	0.995
Intestinibacter_Intestinibacter_bartlettii	0.000	0.039	0.996
Bittarella_Bittarella_massiliensis	0.044	0.075	0.559

Table S12. Correlations between ImP associate bacterial species and *hutH*

ImPA associated species	*hutH* Spearman rho	P_Value
Blautia_hansenii	0.177	0.003
Dysgonomonas_capnocytophagoides	0.034	0.570
Clostridium_scindens	0.021	0.730
Pediococcus_acidilactici	0.018	0.758
Bifidobacterium_reuteri	0.130	0.029
Megasphaera_sp._BV3C161	-0.014	0.819
Citrobacter_freundii	0.087	0.145
Veillonella_parvula	0.237	<0.001
Paraprevotella_xylaniphila	-0.049	0.411
Streptococcus_agalactiae	0.021	0.721
Coprobacter_secundus	-0.137	0.021
Kluyvera_ascorbata	-0.013	0.825
Clostridioides_difficile	-0.200	0.001
Roseburia_hominis	-0.152	0.010
Fournierella_massiliensis	-0.066	0.266
Pseudoflavonifractor_capillosus	-0.214	<0.001
Lachnoanaerobaculum_sp._OBRC55	-0.117	0.049

Table S13 . The presence of *hutH* in specific species : Sequence Alignment analyses

				Enzyme: EC:4.3.1.3; hutH<br>Annotation: histidine ammonia-lyase ;<br>Representive Reference Sequences: hutH;<br>Representive KO group: K01745				Enzyme: EC:1.3.99.33; urdA<br>Annotation: urocanate reductase ;<br>Representive Reference Sequences: urdA<br>Representive KO group: K17363			
species	Association with ImPA	Representative Reference genomes of the species	Correlation with hutH	Presence of hutH	Query Coverage	E value	Percent indentity	Presence of urdA	Query Coverage	E value	Percent indentity
Blautia_hansenii	Pos	*Blautia hansenii DSM 20583*	Pos	Y	100%	0	100.00%	N	8%	0.35	91.30%
Dysgonomonas_capnocytophagoides	Pos	*Reference genomes not available*	NS								
Clostridium_scindens	Pos	*Clostridium scindens ATCC 35704*	NS	N	3%	0.11	88.89%	Y	100%	0	100.00%
Pediococcus_acidilactici	Pos	*Pediococcus acidilactici PMC65*	NS	N	5%	0.062	100.00%	N	5%	0.24	91.30%
Bifidobacterium_reuteri	Pos	*Bifidobacterium longum subsp.JCM 1217*	Pos	Y	100%	0	100.00%	N	20%	0.28	78.95%
Megasphaera_sp._BV3C161	Pos	*Megasphaera sp. BV3C16-1*	NS	N	32%	78%	100%	N	4%	0.069	81%
Citrobacter_freundii	Pos	*Citrobacter freundii complex sp. CFNIH3*	NS	Y	100%	0	100.00%	N	20%	2.2	95.00%
Veillonella_parvula	Pos	*Veillonella parvula DNF00876*	Pos	N	20%	6%	89%	N	6%	9.9	94%
Paraprevotella_xylaniphila	Neg	*Paraprevotella xylaniphila strain YIT 11841*	NS	N	No significant similarity found.			N	No significant similarity found.		
Streptococcus_agalactiae	Neg	*Streptococcus agalactiae strain S25*	NS	N	No significant similarity found.			N	13%	0.21	82.80%
Coprobacter_secundus	Neg	*Coprobacter secundus, Strain 177*	Neg	N	No significant similarity found.			N	No significant similarity found.		
Kluyvera_ascorbata	Neg	*Kluyvera ascorbata ATCC 33433*	NS	N	No significant similarity found.			N	No significant similarity found.		
Clostridioides_difficile	Neg	*Clostridium difficile CD630DERM*	Neg	N	No significant similarity found.			N	24%	0.5	85.71%
Roseburia_hominis	Neg	*Roseburia hominis, A2-183*	Neg	N	No significant similarity found.			N	24%	0.42	80.56%
Fournierella_massiliensis	Neg	*Fournierella massiliensis AM2*	NS	N	No significant similarity found.			N	No significant similarity found.		
Pseudoflavonifractor_capillosus	Neg	*Pseudoflavonifractor capillosus ATCC 29799*	Neg	N	No significant similarity found.			N	12%	0.011	69.52%
Lachnoanaerobaculum_sp._OBRC55	Neg	*Lachnoanaerobaculum sp. OBRC5-5*	Neg	N	No significant similarity found.			N	8%	1.2	77.50%

Supplemental Materials

Supplemental Methods

Study Population

The WIHS was a prospective cohort study of women with or at risk for HIV infection, now continuing as part of the Multi-Center AIDS Cohort Study (MACS)-WIHS Combined Cohort Study, and details on study design and methods have been described previously [1-3]. Every 6 months, WIHS participants undergo a core visit with a comprehensive physical examination, providing biological specimens and completing interviewer-administered questionnaires. Measurements and samples for this study were drawn from the semiannual WIHS clinic visits, each featuring stored blood biospecimens, from return visits to complete the carotid artery ultrasound substudy, and using fecal collection that was performed at home and returned in a specialized mailer. In this study, we included 493 WIHS women whose fecal samples were collected using a home-based self-collection kit [4, 5] during 2017–2019. Among these participants, 320 women underwent carotid artery imaging. We also included 433 women who had proteomic inflammatory markers data on serum samples, and metabolomic/lipidomic data on plasma samples, which were collected during 2017–2019 at the closest WIHS core visit to the time of stool sample collection. The study was reviewed and approved by the institutional review boards at all participating institutions. All participants provided written informed consent.

Shotgun metagenomics sequencing

Metagenomics Sequencing was performed on DNA extracted from fecal samples collected by FTA card using a novel shallow-coverage method of shotgun sequencing-based Illumina NovaSeq platforms [6, 7]. The adapters and barcode indices are processed following the iTru adapter protocol [8]. De-multiplexing was applied to generate Shallow shotgun per-sample FASTQ data and the adapter sequences were trimmed. The human-filtered FASTQ reads were further trimmed to remove low quality bases that had a PHRED quality score of 25 or less. Samples with a coverage depth less than 100,000 reads per sample were excluded. The quality controlled paired end data was then concatenated and aligned against the NCBI RefSeq representative prokaryotic genome collection (release 82) [9] using default SHOGUN [10] settings. Bowtie2[11] was selected as the alignment tools in SHOGUN pipeline. The reads that mapped to a single reference genome is labeled with the NCBI taxonomic annotation at species level. The α-diversity indices (Shannon index, Chao-1 Index and Simpson's Index), and β- diversity weighted UniFrac distances were calculated using Qiita [12] and R phyloseq / vegan packages [13, 14]. Functional components were obtained using SHOGUN and the Kyoto Encyclopedia of Genes and Genomes (KEGG) database release 94.0 [15].

Proteomic Inflammatory markers

Serum proteomics profiling was performed using Olink® Target Inflammation panel (Olink, Boston, Massachusetts). This panel measures 92 immune markers simultaneously. Protein concentrations were reported as normalized protein expression (NPX) units, which are Ct values from the PCR read out and normalized by the subtraction of values for extension control, as well as an inter-plate control. The scale is shifted using a correction factor (normal background noise) and log2 scaled [16, 17]. After quality control, in the current analysis we included 74 inflammatory markers which were detected in >75% of the samples.

Metabolomic/lipidomic profiling

Plasma metabolomic/lipidomic profiling was performed using liquid chromatography-tandem mass spectrometry (LC-MS) at the Broad Institute Metabolomics Platform (Cambridge, Massachusetts). LC affords reproducible separation of metabolites on the basis of their physical properties and MS enables further resolution of metabolites on the basis of mass-to-charge ratio (m/z).Two separate LC-MS methods were performed to measure lipids (lipidomics) and polar metabolites (metabolomics) in each sample, as previously described [18-20] . Raw data from Orbitrap mass spectrometers were processed using Progenesis QI software (NonLinear Dynamics) for feature alignment, untargeted signal detection, and signal integration. Metabolites were quantified using area-under-the-curve of the peaks. The targeted processing of known metabolites was conducted using TraceFinder software (version 3.1, Thermo Fisher Scientific; Waltham, MA). We included a total of 211 lipids and 167 polar metabolites in the current analysis, and all metabolites had coefficient variation <30% and missing rate <20%. Metabolites with missing data (under detectable levels) were imputed with ½ the minimum values for a given metabolite.

Carotid Artery Plaque Ascertainment

High-resolution B-mode carotid artery ultrasound was used to image 8 locations in the right carotid artery of participants: the near and far walls of the common carotid artery, carotid bifurcation, internal and external carotid artery. A standardized protocol was used at each visit by all sites [21, 22].Focal plaque measures were obtained at a centralized reading center (University of Southern California). We defined a focal plaque as an area with localized intima-media thickness of >1.5 mm in any of the 8 imaged carotid artery locations[23].

Assessments of HIV infection and other variables

Demographic, behavioral, clinical, and laboratory variables were collected using standardized protocols at semiannual core study visits[24]. HIV infection was ascertained by enzyme linked immunosorbent assay and confirmed by Western blot. HIV-specific parameters included CD4+ T-cell counts, HIV-1 viral load, and detailed information on specific classes of ART drugs (protease inhibitors, nonnucleoside reverse transcriptase inhibitors, and nucleoside reverse transcriptase inhibitors) [25]. Conventional CVD risk factors included body mass index (BMI), waist to hip ratio, systolic blood pressure(SBP), diastolic blood pressure(DBP), triglycerides, total cholesterol, low-density lipoprotein cholesterol, high-density lipoprotein cholesterol, fasting

glucose ,Hemoglobin A1c, anti-cholesterol and anti-hypertensive medication [26].

Statistical analysis

Gut microbiota (taxa and functional components) and prevalent carotid artery plaque.

We first examined the associations of gut microbiota features with carotid artery plaque among 320 women with corresponding data available. Demographic and clinical characteristics between participants with and without carotid artery plaque were compared using the Mann-Whitney *U* test (continuous variables) and χ^2 test (categorical variables). The Kruskal-Wallis test was applied to compare differences in the microbial α-diversity indices by carotid artery plaque status. Permutational multivariate ANOVA (PERMANOVA) and principal-coordinate analysis (PCoA) were carried out for the microbial β-diversity analyses. Linear discriminant analysis effect size (LefSe)[27] was used to identify gut bacterial species associated with prevalent carotid artery plaque. We then used logistic regression models to examine the multivariate-adjusted associations of LefSe-identified species with carotid artery plaque, adjusting for age, race, study site, antibiotics use, income, education, BMI, alcohol, smoking status, HIV status and ART use. Centered log-ratio (CLR) transformation was conducted for the abundance of taxonomic units before analyses. In addition, we also applied Analysis of Composition of Microbiomes (ANCOM2)[28] to detect gut bacterial species associated with carotid artery plaque, adjusting for aforementioned covariates. We controlled the false discovery rate (FDR) at 10%, and excluded the species if they were present in <20% of the study population or average **relative abundance <0.001%**. The ANCOM detection level ≥0.6 was considered significant which indicates that the ratios of the taxon to at least 60% of other taxa were detected to be significantly different (FDR <0.10) between women with and without carotid artery plaque. Spearman correlation was employed to estimate correlation coefficients among the identified carotid artery plaque-associated species, and traditional CVD risk factors including BMI, SBP, DBP, triglycerides, total cholesterol, low-density lipoprotein cholesterol, high-density lipoprotein cholesterol, fasting glucose and Hemoglobin A1c (participants with anti-cholesterol or anti-hypertensive medication were excluded in the corresponding analyses). We also examined the associations of the plaque-associated species with HIV-specific variables including CD4 count, HIV viral load and current ART treatment using multivariable regression models, adjusting for aforementioned covariates. In addition, to explore potential HIV-specific results, LefSe and logistic regression analyses were also conducted in women with and without HIV infection separately.

For metagenomics functional components analyses, centered log-ratio transformation was applied to the abundance of annotated enzymes. Partial spearman correlation analysis was performed to estimate correlation coefficients between microbial functional enzymes, and plaque related bacterial species. Linear regression models were applied to examine associations of enzymes with plaque, after controlling for the aforementioned covariates. The enrichment test was performed for the 1634 annotated enzymes at EC level III enzyme category, with FDR <0.10 as cut off.

Proteomics inflammatory markers and carotid artery plaque

We examined the associations of Proteomics inflammatory markers with carotid artery plaque among 290 women. Partial least squares discriminant analysis(PLSDA)[29], with the loading

scores of each PLSDA principal components, were used to identify plaque associated proteomics inflammatory markers, and their contribution to each PLSDA principal components. We further performed logistic regression models to examine the associations between carotid artery plaque, and PLSDA principal components and individual inflammatory markers (inverse normal transformed), adjusting for aforementioned covariates. In addition, to explore potential HIV-specific results, logistic regression analyses were also conducted in women with and without HIV infection separately.

Carotid artery plaque-associated microbial species and serum inflammatory markers

We then examined the associations of plaque-associated bacterial species with host serum inflammatory markers (n=426). Spearman correlation was employed to estimate correlation coefficients of identified bacterial species with overall proteomics inflammation profiles estimated by the PLSDA principle components, respectively, as well as individual inflammatory markers.
We further adjusted proteomic profiles in the logistic regressions to examine the potential mediating effect on the association between microbial species and plaque, adjusting for the aforementioned covariates.

Plasma metabolomic profiles, gut microbiome, and serum inflammatory markers, in relation to carotid artery plaque .

In 426 women who had both microbiome and metabolome data, we examined the associations of the 6 plaque-associated bacterial species with plasma lipidomic and metabolomic profiles. Spearman correlation was employed to estimate correlation coefficients of identified bacterial species with individual 211 lipids and 167 polar metabolites, with FDR <0.10 as cut off. Then we used logistic regression models to examine associations between the selected microbial-correlated-metabolites and plaque, adjusting for the aforementioned covariates (n=290). In addition, the stratified analyses were conducted in women with and without HIV infection separately.
We further explored the correlations among these microbial-correlated-metabolites and overall proteomics profiles, as well as individual inflammatory markers (n=433). We further adjusted the microbial metabolites, and proteomic profiles in logistic regressions to examine the potential mediating effect on the associations between microbial species and plaque(n=285), adjusting for the aforementioned covariates.

ImP, ImP related microbial species, functional components, and Serum Inflammatory markers, in relation to Carotid Artery Plaque

To identify the potential gut microbial ImP producer, we first explored the correlations among 316 gut microbial species and plasma ImP level (n=426), with FDR <0.10 as cut off. Then we included all the 138 ImP correlated microbial species in the same conditional analysis regression model (mutual adjustment) after multivariable adjustment, to further examine independent associations between microbial species and ImP.

To better represent the overall GMB features associated with ImP, we calculated a GMB score based on the sum of the weighted INT- CLR transformed abundance of the 17 microbial species independently associated with ImP (weighted by their beta coefficient with ImP in the regression model). Associations of the GMB score and 17 individual microbial species with plasma ImP and plaque status were examined using linear regression models and logistic regression models

respectively, with adjustment for the aforementioned covariates. We then used linear regression to examine associations among levels of functional enzyme hutH, the GMB score (Low,Q1; Medium, Q2 and Q3; High, Q4), and plasma ImP. We also explored the correlations of the GMB score and 17 individual microbial species with functional enzyme hutH (n=426) ; as well as with overall proteomic profiles and individual inflammatory markers (n=433).

The Benjamini-Hochberg false discovery rate (FDR) method was used for the multiple testing correction. Statistical analyses were performed using R 4.0.2. unless otherwise stated.

References

1. Bacon MC, von Wyl V, Alden C, Sharp G, Robison E, Hessol N, et al. The women's interagency HIV study: An observational cohort brings clinical sciences to the bench. Clin Diagn Lab Immunol. 2005;12:1013-1019.

2. Adimora AA, Ramirez C, Benning L, Greenblatt RM, Kempf M-, Tien PC, et al. Cohort profile: The women’s interagency HIV study (WIHS). Int J Epidemiol. 2018;47:393-394I.

3. D'Souza G, Bhondoekhan F, Benning L, Margolick JB, Adedimeji AA, Adimora AA, et al. Characteristics of the MACS/WIHS combined cohort study: Opportunities for research on aging with HIV in the longest US observational study of HIV. Am J Epidemiol. 2021;190:1457-1475.

4. Wang Z, Zolnik CP, Qiu Y, Usyk M, Wang T, Strickler HD, et al. Comparison of fecal collection methods for microbiome and metabolomics studies. Front Cell Infect Microbiol. 2018;8:301.

5. Moon JY, Zolnik CP, Wang Z, Qiu Y, Usyk M, Wang T, et al. Gut microbiota and plasma metabolites associated with diabetes in women with, or at high risk for, HIV infection. EBioMedicine. 2018;.

6. Costello M, Fleharty M, Abreu J, Farjoun Y, Ferriera S, Holmes L, et al. Characterization and remediation of sample index swaps by non-redundant dual indexing on massively parallel sequencing platforms. BMC Genomics. 2018;19:332-018-4703-0.

7. Sanders JG, Nurk S, Salido RA, Minich J, Xu ZZ, Zhu Q, et al. Optimizing sequencing protocols for leaderboard metagenomics by combining long and short reads. Genome Biol. 2019;20:1-14.

8. Glenn TC, Nilsen RA, Kieran TJ, Sanders JG, Bayona-Vásquez NJ, Finger JW, et al. Adapterama I: Universal stubs and primers for 384 unique dual-indexed or 147,456 combinatorially-indexed illumina libraries (iTru & iNext). PeerJ. 2019;7:e7755.

9. O'Leary NA, Wright MW, Brister JR, Ciufo S, Haddad D, McVeigh R, et al. Reference sequence (RefSeq) database at NCBI: Current status, taxonomic expansion, and functional annotation. Nucleic Acids Res. 2016;44:D733-45.

10. Hillmann B, Al-Ghalith GA, Shields-Cutler RR, Zhu Q, Gohl DM, Beckman KB, et al. Evaluating the information content of shallow shotgun metagenomics. mSystems. 2018;3:10.1128/mSystems.00069-18. eCollection 2018 Nov-Dec.

11. Langmead B, Salzberg SL. Fast gapped-read alignment with bowtie 2. Nat Methods. 2012;9:357-359.

12. Gonzalez A, Navas-Molina JA, Kosciolek T, McDonald D, Vazquez-Baeza Y, Ackermann G, et al. Qiita: Rapid, web-enabled microbiome meta-analysis. Nat Methods. 2018;15:796-798.

13. McMurdie PJ, Holmes S. Phyloseq: An R package for reproducible interactive analysis and graphics of microbiome census data. PLOS ONE. 2013;8:e61217.

14. Oksanen J. Multivariate analysis of ecological communities in R: Vegan tutorial. version 2.3-0. 2015;.

15. Kanehisa M, Furumichi M, Tanabe M, Sato Y, Morishima K. KEGG: New perspectives on genomes, pathways, diseases and drugs. Nucleic Acids Res. 2017;45:D353-D361.

16. Berggrund M, Ekman D, Gustavsson I, Sundfeldt K, Olovsson M, Enroth S, et al. Protein detection using the multiplexed proximity extension assay (PEA) from plasma and vaginal fluid applied to the indicating FTA elute micro card™. Journal of Circulating Biomarkers. 2016;5:.

17. Zhang W, Ambikan AT, Sperk M, van Domselaar R, Nowak P, Noyan K, et al. Transcriptomics and targeted proteomics analysis to gain insights into the immune-control mechanisms of HIV-1 infected elite controllers. EBioMedicine. 2018;27:40-50.

18. Qi Q, Hua S, Clish CB, Scott JM, Hanna DB, Wang T, et al. Plasma tryptophan-kynurenine metabolites are altered in human immunodeficiency virus infection and associated with progression of carotid artery atherosclerosis. Clin Infect Dis. 2018;67:235-242.

19. Zhao W, Wang X, Deik AA, Hanna DB, Wang T, Haberlen SA, et al. Elevated plasma ceramides are associated with antiretroviral therapy use and progression of carotid artery atherosclerosis in HIV infection. Circulation. 2019;.

20. Paynter NP, Balasubramanian R, Giulianini F, Wang DD, Tinker LF, Gopal S, et al. Metabolic predictors of incident coronary heart disease in women. Circulation. 2018;137:841-853.

21. Hanna DB, Post WS, Deal JA, Hodis HN, Jacobson LP, Mack WJ, et al. HIV infection is associated with progression of subclinical carotid atherosclerosis. 2015;61:640-650.

22. Hodis HN, Mack WJ, LaBree L, Selzer RH, Liu C-, Liu C-, et al. The role of carotid arterial intima - media thickness in predicting clinical coronary events. 1998;128:262-269.

23. Touboul P-, Hennerici MG, Meairs S, Adams H, Amarenco P, Bornstein N, et al. Mannheim carotid intima-media thickness and plaque consensus (2004-2006-2011). 2012;34:290-296.

24. Hanna DB, Post WS, Deal JA, Hodis HN, Jacobson LP, Mack WJ, et al. HIV infection is associated with progression of subclinical carotid atherosclerosis. Clin Infect Dis. 2015;61:640-650.

25. Kaplan RC, Kingsley LA, Gange SJ, Benning L, Jacobson LP, Lazar J, et al. Low CD4+ T-cell count as a major atherosclerosis risk factor in HIV-infected women and men. 2008;22:1615-1624.

26. Hanna DB, Lin J, Post WS, Hodis HN, Xue X, Anastos K, et al. Association of macrophage inflammation biomarkers with progression of subclinical carotid artery atherosclerosis in HIV-infected women and men. J Infect Dis. 2017;215:1352-1361.

27. Segata N, Izard J, Waldron L, Gevers D, Miropolsky L, Garrett WS, et al. Metagenomic biomarker discovery and explanation. Genome Biol. 2011;12:R60-2011-12-6-r60.

28. Mandal S, Van Treuren W, White RA, Eggesbø M, Knight R, Peddada SD. Analysis of composition of microbiomes: A novel method for studying microbial composition. Microb Ecol Health Dis. 2015;26:27663-27663.

29. Westerhuis JA, Hoefsloot HCJ, Smit S, Vis DJ, Smilde AK, van Velzen EJJ, et al. Assessment of PLSDA cross validation. Metabolomics. 2008;4:81-89.
